# Transcriptome Profiling Reveals Higher Vertebrate Orthologous of Intra-Cytoplasmic Pattern Recognition Receptors in Grey Bamboo Shark

**DOI:** 10.1371/journal.pone.0100018

**Published:** 2014-06-23

**Authors:** Tirumurugaan Krishnaswamy Gopalan, Pradheepa Gururaj, Ravi Gupta, Dhinakar Raj Gopal, Preeti Rajesh, Balachandran Chidambaram, Aravindan Kalyanasundaram, Raja Angamuthu

**Affiliations:** 1 Department of Animal Biotechnology and Translational Research Platform for Veterinary Biologicals, Tamil Nadu Veterinary and Animal Sciences University, Chennai, Tamil Nadu, India; 2 Department of Animal Biotechnology, Tamil Nadu Veterinary and Animal Sciences University, Chennai, Tamil Nadu, India; 3 SciGenom Labs Pvt. Ltd., Kakkanad, Cochin, Kerala, India; 4 Department of Veterinary Pathology, Tamil Nadu Veterinary and Animal Sciences University, Chennai, Tamil Nadu, India; Uppsala University, Sweden

## Abstract

From an immunologist perspective, sharks are an important group of jawed cartilaginous fishes and survey of the public database revealed a great gap in availability of large-scale sequence data for the group of Chondrichthyans the elasmobranchs. In an attempt to bridge this deficit we generated the transcriptome from the spleen and kidney tissues (a total of 1,606,172 transcripts) of the shark, *Chiloscyllium griseum* using the Illumina HiSeq2000 platform. With a cut off of > = 300 bp and an expression value of >1RPKM we used 43,385 transcripts for BLASTX analysis which revealed 17,548 transcripts matching to the NCBI nr database with an E-value of < = 10^−5^ and similarity score of 40%. The longest transcript was 16,974 bases with matched to HECT domain containing E3 ubiqutin protein ligase. MEGAN4 annotation pipeline revealed immune and signalling pathways including cell adhesion molecules, cytokine-cytokine receptor interaction, T-cell receptor signalling pathway and chemokine signaling pathway to be highly expressed in spleen, while different metabolism pathways such as amino acid metabolism, carbohydrate metabolism, lipid metabolism and xenobiotic biodegradation were highly expressed in kidney. Few of the candidate genes were selected to analyze their expression levels in various tissues by real-time PCR and also localization of a receptor by *in-situ* PCR to validate the prediction. We also predicted the domains structures of some of the identified pattern recognition receptors, their phylogenetic relationship with lower and higher vertebrates and the complete downstream signaling mediators of classical dsRNA signaling pathway. The generated transcriptome will be a valuable resource to further genetic and genomic research in elasmobranchs.

## Introduction

The cartilaginous fishes (Chondrichthyans) include two major groups namely the holocephalans (chimaeras with fixed upper jaw) and elasmobranchs (includes skates, rays and sharks with protractible upper jaw) that are interesting, phylogenetically important and oldest group of living vertebrates [1]. They have been evolving independently after they diverged from a common ancestor460–520 million years ago and hence represent distinct lineages [2]. In this context, among the extant animals, studies on the chondrichthyans considered to be the ancestors of the jawed vertebrates are important and would provide new insights in understanding various aspects of evolution of complex vertebrate systems and their applications in modern biology [3]. Significant research on various aspects has been carried out with the small sharks to gain more understanding on this species [4]. Cartilaginous fishes including sharks have also evolved unique physiological adaptations to survive in marine environment [5]. Evidence does point to the development of specific immune responses evolved from this species and hence sharks are considered as a species of “immunologist's delight”. With the receptor evolution even apparent among jawed vertebrates, the cartilaginous fishes prove to be a striking dichotomy with respect to immune receptors [6], [7], [8].

Sequence database of non-model organisms have effectively been expanded using next-generation sequencing (NGS) technologies [9], [10]. Commercially available NGS technologies produce high throughput short reads than the Sanger method [11], [12]. This method of transcriptome information generation has several advantages as it is not only fast and cost effective but also detects low-abundance transcripts and generates an extraordinary depth of short reads. Accurate assembly of short reads generated in the NGS platforms are essential in sequence annotation [13] and transcriptome analysis of non-model organisms [10], [14]. Over 100 studies have been conducted on gene expression pattern, single nucleotide polymorphism (SNP) identification, transcriptome analysis involving various aquaculture species, using the NGS platforms [15] as well as on the complete set of all transcripts from certain types of cells or tissues [16], [17]. Recent transcriptome studies on soft-shelled turtle, basal jawed vertebrates and neo-tropical catfish [18], [19], [20]; provide evidence for suitability of this platform for surveying the complex vertebrate transcripts. Despite these advancements, transcriptome studies in sharks are still limited to only hagfish shark embryos [21]. There are only 8 nucleotides (nt) and 2366 expressed sequence tags (EST) of the grey bamboo shark *Chiloscyllium griseum* available at NCBI and as on date there is no report on the grey bamboo shark *Chiloscyllium griseum* EST generated using [NGS).

Hence, we made preliminary attempts to generate the whole transcriptome of *Chiloscyllium griseium* using Illumina HiSeq2000 platform from two of the important organs, namely spleen and kidney. A comprehensive functional annotation of the assembled transcriptome is performed in this study. We validated the annotated data by determining the phylogenetic relationship and expression profile across different tissues by quantitative real-time PCR (qRT-PCR) for some of the annotated and identified sequences from both the organs. Several transcripts expressed particularly at higher levels in spleen and kidney was also identified. To validate our bioinformatic analysis, we have amplified a full-length gene; sequence characterized, localized and profiled this transcript to different tissues, and identified the downstream mediators to this transcript from the transcriptome to indicate the existence of this pathway

## Materials and Methods

### Ethics Statement

The study was not carried out in any national parks or protected areas. *Chiloscyllium griseum* (Muller and Henle 1838) is widely distributed in the coastal regions of Tamil Nadu, India was selected for this study as they are not a schedule I species under Indian Wildlife Protection Act, 1972. In India experimentation with fishes in general including sharks is not under the purview of the Institutional Animal Ethical Committee (IAEC).

*C. griseum* are regularly taken in inshore fisheries off Pakistan, India and Thailand, and utilized for human food (http://www.fao.org/fishery/species/11342/en) and such sharks collected by deep fishing were obtained from the local fisherman to be maintained in a simulated and controlled aquarium facility at Tamil Nadu Veterinary and Animal Sciences University, Chennai, India in the project funded by the ICAR-NAIP (C30018) (http://www.fish-tlr-naip.in/web/). Ethylene glycol monophenyl ether (Sigma, USA) @ 5 ml/L of water was used for anaesthesia and following deep anaesthesia the organs were collected for the study

### 1. *Chiloscyllium griseum* (Grey Bamboo Shark)

*Chiloscyllium griseum* (Muller and Henle 1838) a sluggish, bottom dweller shark widely distributed in the coastal regions of Tamil Nadu, India was selected for this study as they are not a schedule I species under Indian Wildlife Protection Act, 1972. In India experimentation with fishes in general including sharks is not under the purview of the Institutional Animal Ethical Committee (IAEC). *C. griseum* are regularly taken in inshore fisheries off Pakistan, India and Thailand, and utilized for human food (http://www.fao.org/fishery/species/11342/en) and such sharks collected by deep fishing were obtained from the local fisherman to be maintained in a simulated and controlled aquarium facility at Tamil Nadu Veterinary and Animal Sciences University, Chennai, India in the project funded by the ICAR-NAIP (C30018) (http://www.fish-tlr-naip.in/web/). Ethylene glycol monophenyl ether (Sigma, USA) @ 5 ml/L of water was used for anesthesia and following deep anaesthesia, the spleen and kidney were excised, flash frozen in liquid nitrogen and shipped on dry ice to SciGenom Labs, Cochin for further processing.

### 2. RNA isolation, library preparation and sequencing

Total RNA was isolated from spleen and kidney tissues (n = 2 animals) using PureLink RNA mini kit (Ambion, TX) and quality assessed using Qubit 2.0 Fluorometer using the Qubit RNA BR Assay Kit(Life Technologies, CA) and on a 2100 Bioanalyzer using an Agilent RNA 6000 Pico Kit(Agilent Technologies, CA). The RIN values were 8.1 and 7.6 respectively for RNA from spleen and kidney indicating that the RNA quality was good for library preparation. cDNA libraries was prepared using the TruSeq RNA Sample Preparation Kit v2low-throughput protocol (Illumina Inc., CA) according to the manufacturer's instructions. The quality of the cDNA library was assessed on a 2100 bioanalyzer using a DNA 1000 kit (Agilent Technologies, CA), concentration measured using library quantification kit (Kapa Biosystems, MA) and sequenced using the HiSeq2000 platform (Illumina Inc., CA) after indexing the samples from the two animals.

### 3. Assembly of transcripts, annotation and differential transcript analysis

Briefly, the transcripts were assembled using SOAP denovo-Trans program [22]. To calculate the expression value, we first aligned the reads to the assembled contigs using Bowtie program [23] allowing upto 3 mismatches and then calculated the RPKM value [24] using custom Perl script. The assembled transcripts were filtered based on minimum length (> = 300 bp) and expression value (> = 1 RPKM). After filtering, the assembled transcripts were annotated using BLASTX program against non-redundant (nr) NCBI database [25], [26]. Annotation was also performed against UniProt database based on BLASTX hits. The annotated unigenes were also mapped to gene ontology (GO) and eggNOG terms. KEGG pathway [27] annotations was performed using MEGAN4 [28] program on the BLASTX hits. To determine the differential (higher) levels of transcripts across kidney and spleen an index file is generated from the assembled transcriptome using Bowtie-build command [23] and the paired-end reads from Kidney and Spleen are mapped to the reference assembled transcriptome index sequence using Bowtie program. The total reads mapped using Bowtie to each transcript is calculated using in-house perl script. The DESeq bio conductor package [29] is then used for identifying the differentially (higher levels) transcript levels between kidney and spleen (the package applies a negative binomial distribution based statistical test to perform the differential expression analysis). The transcripts with p-value < = 0.01 and expression > = 1 FPKM in one of the tissue is scored as differential/highly expressed transcript.

### 4. Phylogenetic analysis and Domain prediction

From the KEGG pathway annotation we short listed the transcripts that were interestingly annotated to immune related function (from spleen and kidney) and also transcripts that were annotated for a role in salt balance and excretion (from kidney). The identified transcripts (TLR3, TLR9, TLR2, TLR6, NOD1, NOD2, RIG-I, MDa5, and the Urea transporter) were analysed for their phylogenetic relationship and also to determine their divergence employing the MEGA software [30]. The GenBank accession numbers of the sequences used for comparison and to determine the phylogenetic relationship in this study are listed in the (Table S1). Multiple alignments of the nucleotide sequences of the identified transcripts with the corresponding sequences from GenBank were performed using ClustalW (codons) in MEGA 6.0, the maximum likelihood fits for the data specific model was used to develop the phylogenetic tree and also to determine their relationship based on the BIC, maximum likelihood values and AICc scores. The percent identity and diversity of the respective nucleotide and protein sequences were determined using the MegAlign module of Lasergene V7.1 (DNASTAR Inc., CA). The deduced amino acids of the above transcripts were also used to predict their domains using the simple modular architecture research tool (SMART) available at http://smart.embl-heidelberg.de/ which confirmed the annotation and identity of the proteins.

### 5. Characterization of dsRNA sensing TLR signaling pathway

The identified TLR3 was sequenced, ORF predicted, phylogenetic relationship determined and the domains predicted. Using the ectodomain of the putative shTLR3 protein sequence and the human TLR3 ECD protein structure (3ULU) were performed homology modelling. Blastp (version 2.2.17) was employed to find the best templates from Protein Data Bank (PDB) using default parameters to download the template structures. The MODELLER 9v7 was used for homology modelling that produced 3D optimized models through molecular probability density function (molpdf). The models were evaluated with 5 different tools namely Verify 3D, Procheck, ERRAT, WHATIF server and ProQ and considered ‘good’ only when it satisfied the threshold criteria in atleast 3 of the tools tested. The structure of the synthetic analog of dsRNA, Poly I:C (CID: 32744) to TLR3 was obtained from PubChem compound database, docked with the generated models using Autodock Vina 1.1.5 and the analysis of docking simulations were carried out using AutoDock Tools. The receptor-ligand complex analysis was performed using Molegro molecular viewer and DS Visualizer.

We also determined the basal expression levels of TLR3 in spleen, kidney, liver, intestinal spiral valve and epigonal tissues and also to localize the shTLR3, in these tissues by *in-situ* PCR. For *in-situ* localization the above mentioned tissues were fixed in 10% formalin, sectioned into 5 µm thick sections and placed on a super frost plus slide (Thermo Scientific). The sections were processed for localization of the TLR3 in different tissue following the procedure described in our earlier report [31]. The classical downstream signaling molecules of the TLR3 signaling pathway namely TRAF3, IRF3, IRF7, TBK1 and Mx were also identified in the annotated data. The downstream mediators were also sequence characterized with respect to their identity, ORF, divergence and phylogenetic relationship with higher vertebrate and piscine sequences as performed for the other transcripts (section 2.4).

### 6. Gene validation and expression analysis

Validation of the sequencing and computational analysis of the generated transcriptome data was performed by experimental confirmation on selecting targets with a wide range (1 to 19) of RPKM value. Three of the genes relating to immune response (TLR9, TLR3 & IL-8) and two involved in osmoregulation (aquaporin & urea transporter) were assessed for the basal transcript levels by qRT-PCR (Primers shown in Table S2). For each target gene reaction were run in triplicate with appropriate no-template controls (n = 3 sharks), internal control (18S rRNA) and RNA controls (to determine DNA contamination). The Ct values were recorded only when NTCs showed no amplification and the data analyzed as described in our earlier study [31].

## Results

### 1. *De novo* transcriptome assembly

The bioinformatics workflow applied in our study to analyse the generated reads is depicted in Figure S1. The sheared poly (A)+RNA from spleen and kidney were isolated, converted to cDNA and sequenced inIllumina HiSeq 2000 that produced 94,538,693 and 93,759,234 raw reads respectively with a read length of 101×2 (paired end reads) generating almost ∼19.1 and ∼18.9 giga bases (∼38 Gb) of raw data respectively. The low quality bases and read portion showing specific base bias were also trimmed from the raw reads. Overall the first 20 bases are trimmed off each read and the trim read sequences (> = Q30% is ∼90.5) from both tissues are concatenated to generate a reference transcriptome for grey bamboo shark. Transcriptome assembly was performed using SOAP denovo-Trans program (*k*-mer = 31). The SOAP denovo-Trans program produced a total of 1,606,172 transcripts. The assembled transcript length and its GC distribution are shown in Figure S2. The mean length of the assembled data is∼180 bp with almost 171,449 transcripts (10.7%) being > = 300 bp in length.The longest transcript identified is of length 16,974 bp. Further, the transcripts showing low expression value (<1 RPKM) are filtered to obtain a final count of 43,385 filtered transcripts (Table 1). The paired end reads generated in this study has been submitted to EBI-ENA under the study PRJEB4999.

**Table 1:** Summary of bamboo shark transcripts generated, assembly and annotation.

Total number of reads (Spleen)	94,538,693
Total number of reads (Kidney)	93,759,234
Total Nucleotides (Gb)	∼38
Total base > = Q30 (%)	∼90.5
GC percentage (%)	∼44.6
Total number of transcripts	1,606,172
Mean length of transcripts (bp)	180
Longest transcript length (bp)	16,974
Total filtered transcripts	43,385
Total filtered transcripts with significant BLASTX hit	17,548
Total filtered transcripts with UniProt Annotation	8,716

### 2. Transcriptome annotation

**Figure 1:**
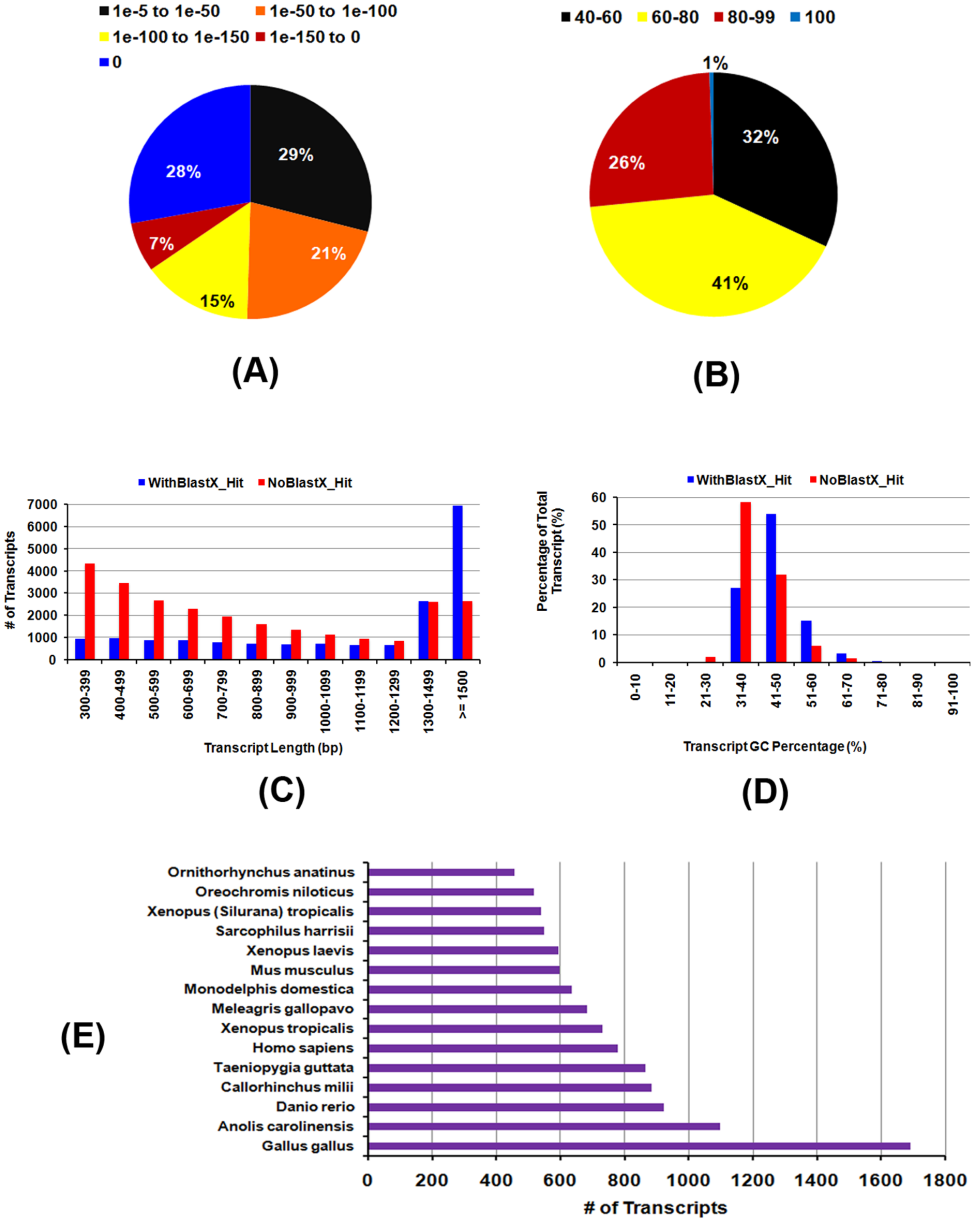
Transcript annotation: *Chiloscyllium griseium* transcripts that revealed BlastX matches to NCBI database. **A & B**- E-value and distribution of the similarity scores; **C&D**- Length and GC distribution of the transcripts that revealed matches and no matches to NCBI database; **E**- BlastX top hit species matches of the *C. griseum* transcripts identified in this study.

The assembled transcripts are compared against the NCBI non-redundant (nr) protein database using BLASTX program. Out of total 43,385 transcripts (Table S3), 17,548 (40.5%) transcripts revealed significant BLASTX matches with E-value of< = 10^−5^ and similarity score > = 40% (Table S4). More than 70% of the BlastX hit transcripts have confidence levels of at least 1E-50 and 68% of the assembled transcripts have a similarity of more than 60% at protein level with the existing proteins at NCBI database. The longest transcript with ID = 3212109 revealed BLASTX hit matches to HECTD4 gene that encodes HECT domain containing E3 ubiqutin protein ligase (NCBI Acc. No. 410047398 & Protein ID - XP_003314026.2 of *Pan troglodytes*). Based on the BLASTX hits, the organisms name for the top hit were extracted and the top 15 organisms is listed in Figure 1 which included matches to *Gallus gallus* (1715 transcripts −9.77%), *Xenopus tropicalis* (1271 transcripts –7.24%), *Anolis carolinensis* (1098 transcripts −6.26%), *Danio rerio* (922 transcripts – 5.25%), *Callorhinchus milli* (915 transcripts – 5.21%), *Taeniopygia guttata* (865 transcripts– 4.93%) and *Homo sapiens* (786 transcripts – 4.48%) (Table S5). The identified 915 transcripts matching to *Callorhinchus milli* (ghost shark) are listed in Table S6.Among the top hits, 11sequences matched to *Chiloscyllium griseum*, which includes NADH dehydrogenase genes (ND1, ND2, ND3, ND4, ND5), cytochrome (COX1, COX2, COX3, CYTB), and ATP synthase F0 subunit 6 (ATP6). It is interesting to note that, out of the 13 gene sequence information available on *C. griseum at* NCBI, we could detect 11 sequences in our transcriptome data. We further annotated the transcripts with BLASTX hits against UniProt database. A total of 8,716 transcripts matched to already reported sequences in UniProt. The transcripts with complete UniProt annotation including protein named and ID, organism name, Gene ontology (GO) and eggNOG annotation, pathway, protein localization and other relevant information are provided in Table S7, Table S8 & Table S9.

### 3. Functional annotation

**Figure 2:**
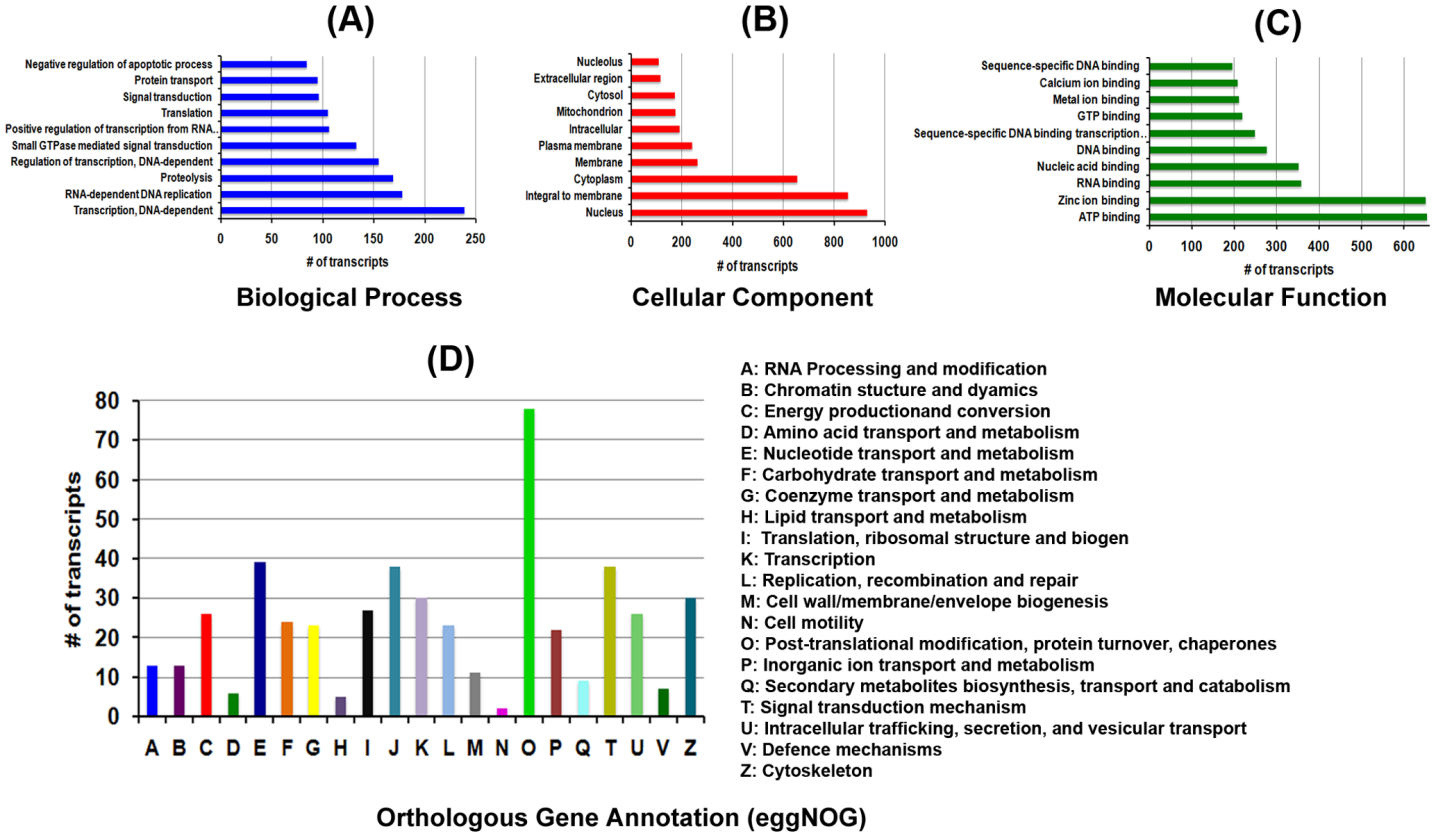
Functional annotation of number of the *Chiloscyllium griseium transcripts with* BlastX matches to different GO category. **A**- Biological process; **B**- Cellular component; **C**- Molecular function; and D-The *C. griseium* transcripts with matches to reported orthologous genes using eggnog.

**Figure 3:**
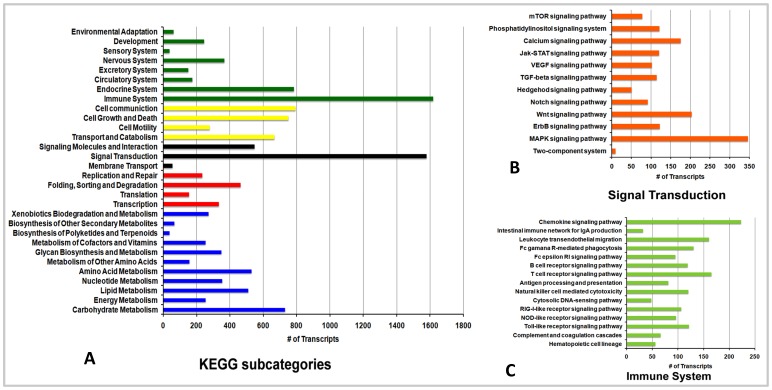
MEGAN4 based mapping of *Chiloscyllium griseium* transcripts to different KEGG pathways. **A** - shows the transcripts grouping to different KEGG sub-categories; **B** – Subcategories of the transcripts grouping to signal transduction pathway and **C** - Subcategories of the transcripts grouping to immune system pathways.

Functional classification of transcripts was performed using GO, eggNOG and KEGG analysis. In total 5,551 different GO terms were identified. Of which 3625, 648 and 1278 transcripts group themselves into biological process, cellular components and molecular function categories respectively Table S8. The top 10 frequent GO terms identified in each category are shown in Figure 2A, 2B and 2C respectively. The orthologous gene annotation (eggNOG) classified the transcripts into 21 major groups and the transcript mapping to each group is shown in Figure 2D, with the details in Table S9. The transcripts with BLASTX were also mapped to KEGG functional classes using MEGAN4 that classified the genes into six different KEGG pathway categories. The KEGG main categories are further sub-classified into several subcategories. The total number of transcripts mapping into the sub-categories is shown in Figure 3A. It clearly indicates that signal transduction and immune system related genes were highly represented in the transcriptome data. Further distribution of total transcripts in signal transduction and immune system related pathway is shown Figure 3B and 3C respectively. From the KEGG pathway annotation we short listed the transcripts that were interestingly annotated to immune related function (from spleen and kidney) Table S10 and Table S11.

### 4. Basal transcript expression level difference (RPKM) across spleen and kidney

**Figure 4:**
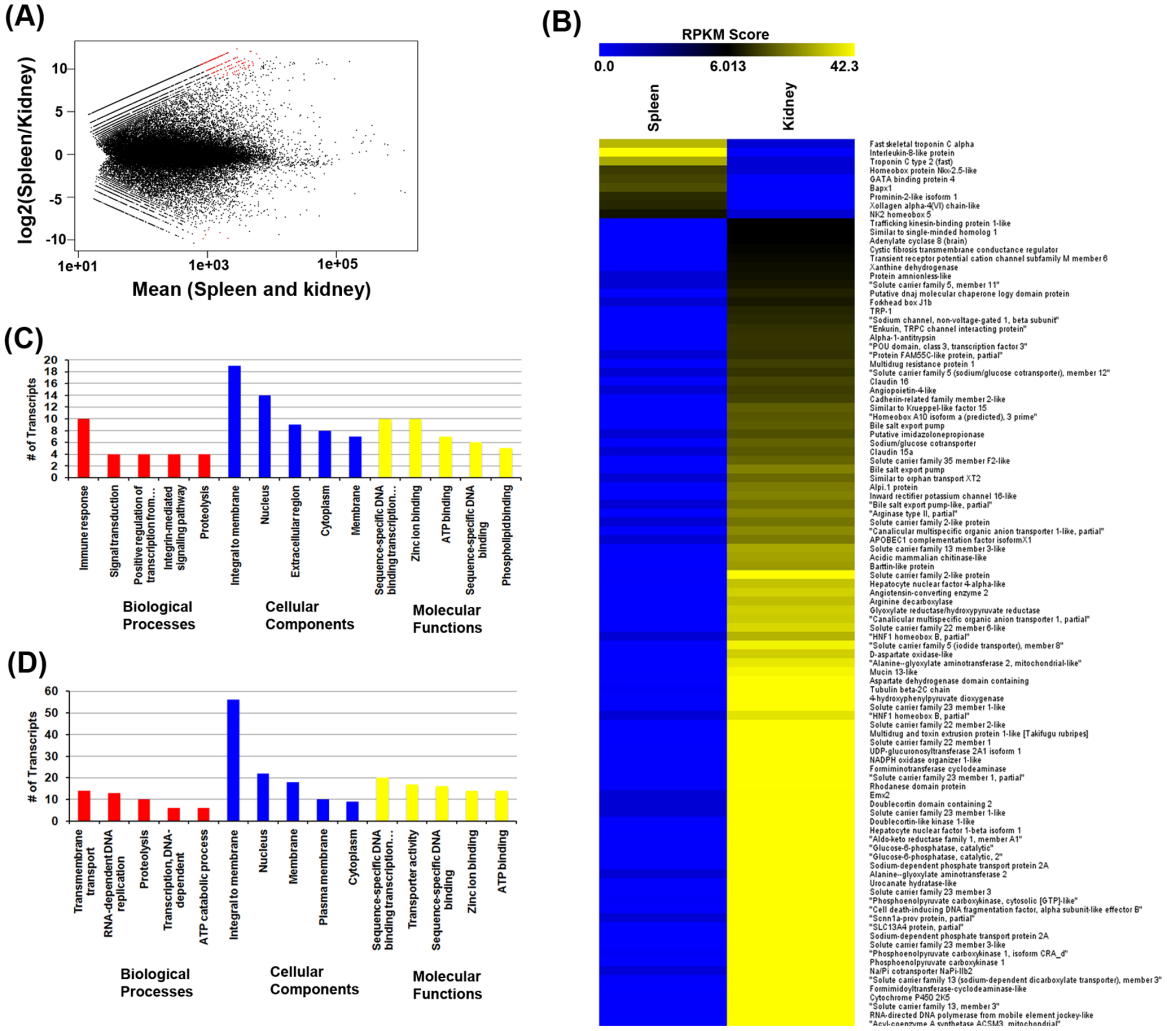
Differential gene expression analysis. **A**- DESeq scatter plot depicting the differentially expressed transcripts across spleen and kidney tissues; **B**- list of the different transcripts across spleen and kidney; Gene ontology of the differentially expressed transcripts in spleen (C) and Kidney (D). *Note the increased representation of the transcripts to immune system in spleen*.

There were 1,881 transcripts that were expressed at higher levels (p-value < = 0.01) across spleen or kidney. The DESeq scatter plot is shown in Figure 4A. The red dots show the differential levels of the transcripts across these two tissues. Of these identified transcripts, there were 695 transcripts expressed at higher levels in spleen (Table S12), and 1,186 transcripts expressed higher in kidney (Table S13). The transcripts that are expressed at higher levels in both these tissues with their BLASTX annotation is shown in Figure 4B. Several transcripts expressed at higher levels could be identified between the two tissues. The ILK-8, Troponin C, NK2 homeobox 5 are expressed at higher levels in spleen, whereas, Na/Pi co-transporter, solute carrier family genes, homeodomain gene Emx2, Alanine-glyoxylate aminotransferase 2, HNF1 hemeobox B are expressed at higher levels in the kidney tissue. Gene ontology revealed transcripts relating to immune system (TNFSF13B, SCYA107, CCL19, ILK-8, CXCR5, MIP3, CD154) are highly expressed in spleen (Figure 4C), whereas a number of highly expressed transcripts in kidney belonged to solute carrier family (slc13a3, slc5a2, slc5a11, slc5a12, slc13a4) (Figure 4D). In order to get more insight into the different levels of these transcripts we used MEGAN4 annotation pipeline and generated the tag cloud plot Figure S3. The highly expressed transcripts in spleen are enriched with immune and signalling pathways including cell adhesion molecules, cytokine-cytokine receptor interaction, T-cell receptor signalling pathway and chemokine signaling pathway.

We could also demonstrate the presence of genes coding for the different toll-like receptor (TLR) types, RIG-I-like receptors (RLRs), different NOD-Like receptors (NLRs). The transcripts showing higher expression in kidney are mostly related to different metabolism pathways such as amino acid metabolism, carbohydrate metabolism, lipid metabolism and xenobiotic biodegradation. It is interesting to note that even though majority of the differentially expressed transcripts under signal transduction are expressed at higher level in spleen, the transcripts homologous to low density lipoprotein (LDL) receptor-related protein 2 or Megalin are highly expressed in kidney. Some of the interested transcripts have been submitted with the accession number listed in Table S14

### 5. Phylogenetic Analyses

Annotation of the transcripts following BLASTX matches revealed a number of cognate genes involved in immune related functions and salt balance. Immunity in invertebrates and lower vertebrates is attributed to the highly conserved innate immune system which uses pathways of regulation and transcription involving homologous receptors and antibacterial peptides, as observed in mammals. Since our earlier report [31] indicated the presence of TIR domain of TLR2 in this species we screened for transcripts that revealed matches to toll-like receptor orthologous.

**Figure 5:**
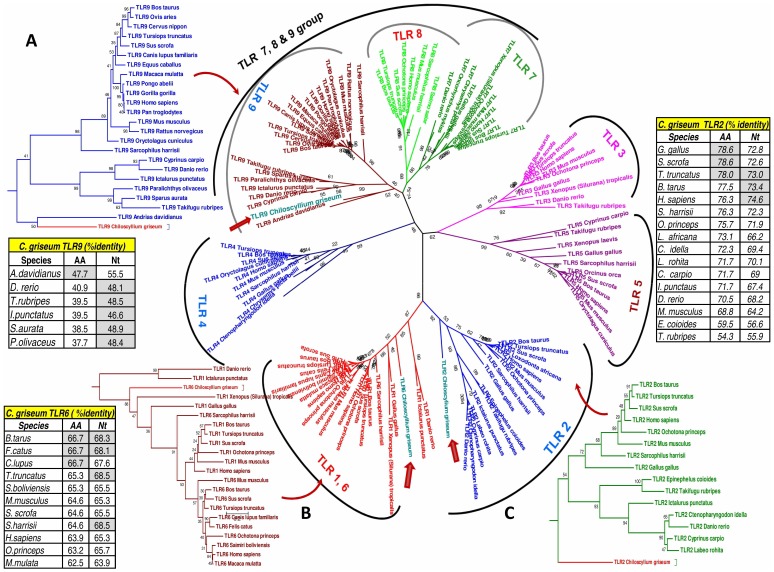
Phylogenetic relationship of the toll like receptor type transcripts of *Chiloscyllium griseum* identified from the transcriptome data. The sequences of the TLR types TLR2 (1410 nt), TLR6 (2436 nt) and TLR9 (1431 nt) of *C. griseum* was aligned with the other sequences as listed in the Table S1 from GenBank representing the TLR types from different lower and higher order species using Clustal W (codons) algorithm in MEGA 5.0. The corresponding nucleotide and amino acid identity of the *C. griseum* transcript is provided in the insert table for each TLR type ***Note***: The grouping of the TLR2 and TLR6 of *C. griseum* with the bacterial PAMP recognizing TLR group (namely the TLR1, 2, 4, 5 & 6) while the TLR9 groups itself with the TLR7, 8 & 9 family.

**Figure 6:**
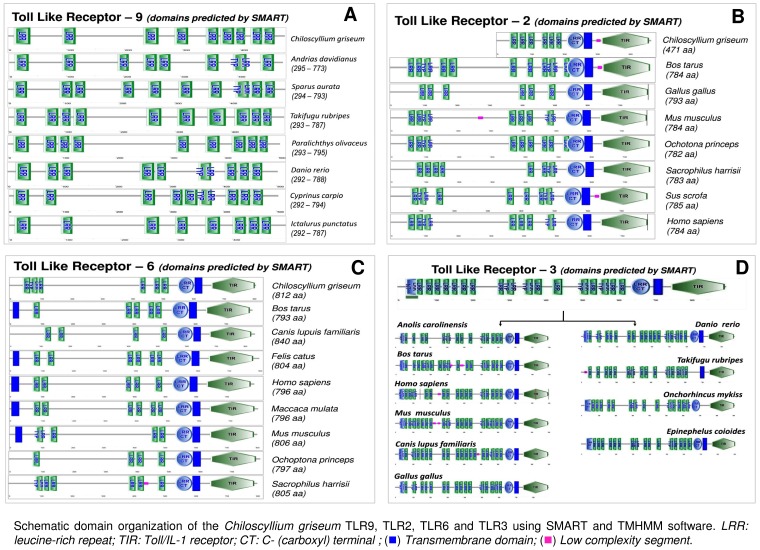
Schematic diagram of the domain organization of the *Chiloscyllium griseum* TLR type transcripts - TLR9, TLR2 TLR6 and TLR3 predicted using the SMART software. **A**– TLR9 domain structure; **B**- TLR2 domain structure; **C** – TLR6 domain structure and **D**- TLR3 domain structure. LRR- luecine rich repeat; TIR – Toll/IL-1 receptor. The domain structure of these TLR types almost remained similar to the higher vertebrate TLRs.

The KEGG pathway analysis also confirmed the presence of the TLR types (TLR2, TLR3, TLR6 and TLR9), NLR types (NOD-1, NOD2), RIG types (RIG-I and MDA5) and also majority of the downstream signalling mediators of these pathways in the transcriptome data (Figure S4A, S4B, S4C & S4D). We selected transcript (Transcript ID -3176873) coding for 477 amino acids which revealed matches with ectodomain region of TLR9 (*Andrias davidianus*); a 470 amino acid sequence (coded by Transcript ID 3183871) revealed matches to TLR2 (*Homo sapiens*), a 900 amino acid sequence which revealed matches to TLR3 (coded by Transcript ID-3207159) *Rattus norvegicus* and a 812 amino acid sequence (coded by Transcript ID 3205147) that revealed matches to the full-length protein sequence of TLR 6 (*Sacrophillus harrisii*) for further analysis. We used the sequences to construct a maximum likelihood tree to infer their phylogenetic relationship with the sequences of the toll-like receptors of lower and higher vertebrates. As expected the un-rooted tree formed six major families, each family marked with unique colour (Figure 5).The TLR9 of *C. griseum* grouped in the TLR7,8 & 9 (family referred based on the lowest ordinal) as well as grouping itself with the TLR 9 sequences of other lower vertebrates. The TLR 9 of *C. griseum* is closely related with the TLR 9 protein of *Andrias davidianus*, the Chinese giant salamander (47.7% aa identity) the existing largest amphibian. The TLR2 and TLR6 sequences grouped themselves in the bacterial ligand recognition group (which includes TLR types TLR1, 2, 6, 4 & 5). The TLR6 protein revealed similarity (66.7% aa identity) with the TLR6 protein of *Bos tarus and Felis catus* while, the TLR2 protein revealed almost maximum similarity (78.6% aa identity) with the TLR2 protein of the higher order vertebrates the *Sus scrofa* and *Gallus gallus*. Domains predicted on the TLR9 protein of *Chiloscyllium griseum* with SMART revealed the protein matching to the position 293 to 787 of TLR9 of different species (Figure 6A). This part of the protein corresponded to the ectodomain of TLR9 due to the presence of 8–9 luecine rich repeat (LRR) domain as seen in the higher order species compared. In the case of TLR 2 the domain prediction also revealed 5 LRR domains in the region compared as also observed with other species (Figure 6B). The domains of the TLR6 also did not reveal many changes in the number of LRR but the length of the TLR6 was 812 amino acids on the contrary to the ∼800 amino acids in most of the animal species compared (796–804 aa) (Figure 6C).

**Figure 7:**
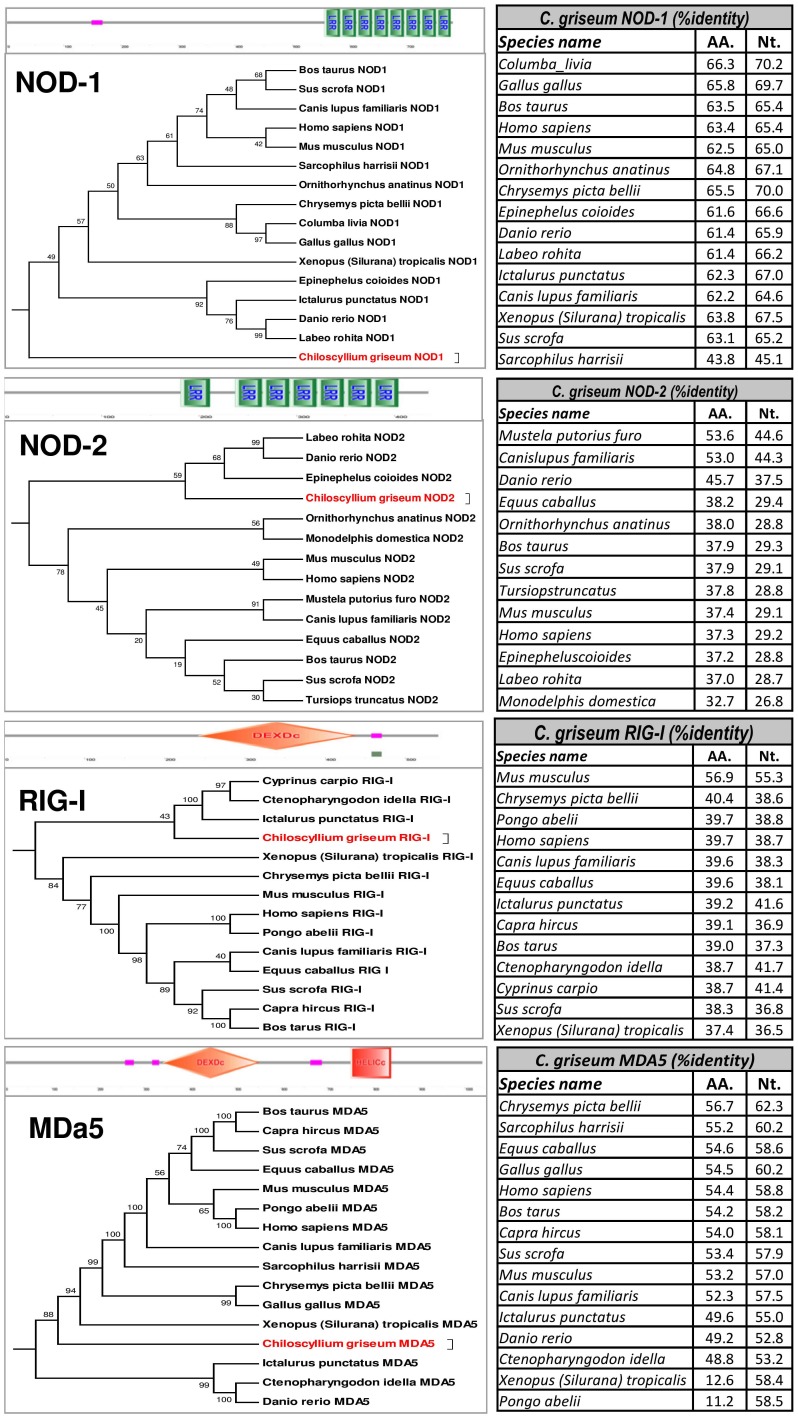
SMART domain prediction and percentage identity of the intra-cytoplasmic pattern recognition receptor transcripts of *Chiloscyllium griseum* identified from the transcriptome data – NOD1, NOD2, RIG-I and MDA5. The sequences of the receptors NOD1 (2838 nt), NOD2 (1308 nt), RIG-I (1599 nt) and MDA5 (3117 nt) of *C. griseum* was used to predict the domains. The identity of these receptors ranged from 53.6% (NOD2), MDA5 (56.7%), 56.9% (RIG-I) and 66.35 (for NOD1). The corresponding nucleotide and amino acid identity of the *C. griseum* transcripts is also provided for each of the receptor type with some of the important species (further details in Table S13).

With respect to the intra-cytoplasmic pattern recognition receptors, the transcript (Transcript ID -3207963) coding for 946 amino acids revealed matches to NOD-1 (*Columba livia*- 66.3% aa identity); a 436 amino acid sequence (coded by Transcript ID 3184971) that exhibited matches to NOD-2 (*Mustela putorius furo*- 53.6% aa identity); a 533 amino acid sequence (coded by Transcript ID 3193515) matching to RIG-I (*Mus musculus*- 56.9% aa identity) and a 1039 amino acid sequence (coded by Transcript ID 3209733) that revealed matches to full-length MDA5 (*Chrysemyl picta bellii* – 56.7% aa identity) (Figure 7).

Being a marine organism it has been shown that urea levels in the body fluids and tissues are as high as 350–600 mM in elasmobranch fishes with remarkable complexity of the kidney nephron structure. We identified a transcript (Transcript ID -3154975) that coded for 92 amino acids and annotated to urea transporter (UT) and generated a maximum likelihood tree to infer its phylogenetic relationship. The UT amino acid sequence of *C. griseum* grouped itself with the UT sequences of other elasmobranch fishes with varied identity (66.3 to 90.2% identity). The greatest similarity of the UT of *C. griseum* was with that of *Triakis scyllium* (90.2%) (Figure S5).

### 6. Characterization of the dsRNA sensing TLR3 in *C. griseum*

**Figure 8:**
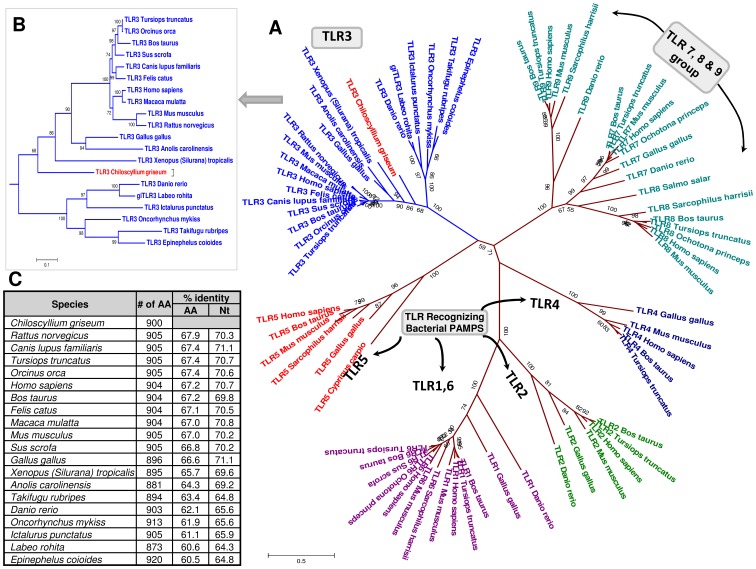
Phylogenetic relationship of the *Chiloscyllium griseum* Toll-like receptor 3 (shTLR3) with the TLR sequences of other species. The full-length coding sequence of the shTLR3 (2703 nt) of *C. griseum* was aligned with the other sequences representing the TLR types from different lower and higher order species using Clustal W (codons) algorithm in MEGA 5.0. The un-rooted shows the grouping of the TLR types in to six major families. The corresponding nucleotide and amino acid identity of the shTLR3 is provided in the insert table *Note*: The shTLR3 grouped with the TLR3 family and revealed close relationship with the higher vertebrate TLRs rather than the teleost TLR3.

Amplification and sequencing the annotated TLR3 revealed an open reading frame of 2703 bases that coded for 900 amino acids with a predicted signal peptide region of 23 amino acids (Data S1). The TLR3 sequence was more related to higher vertebrate rather than piscine (Figure 8). Comparison revealed greater identity at amino acid and nucleotide level to *Rattus norvegicus* (67.9 and 70.3% respectively) and *Canis lupus familiaris* (67.4 and 71.1% respectively). SMART analysis of the TLR3 protein revealed a typical TLR architecture in the ectodomain (ECD) with 19 LRR domains (including N & C-terminal LRR), transmembrane and TIR domains.

**Figure 9:**
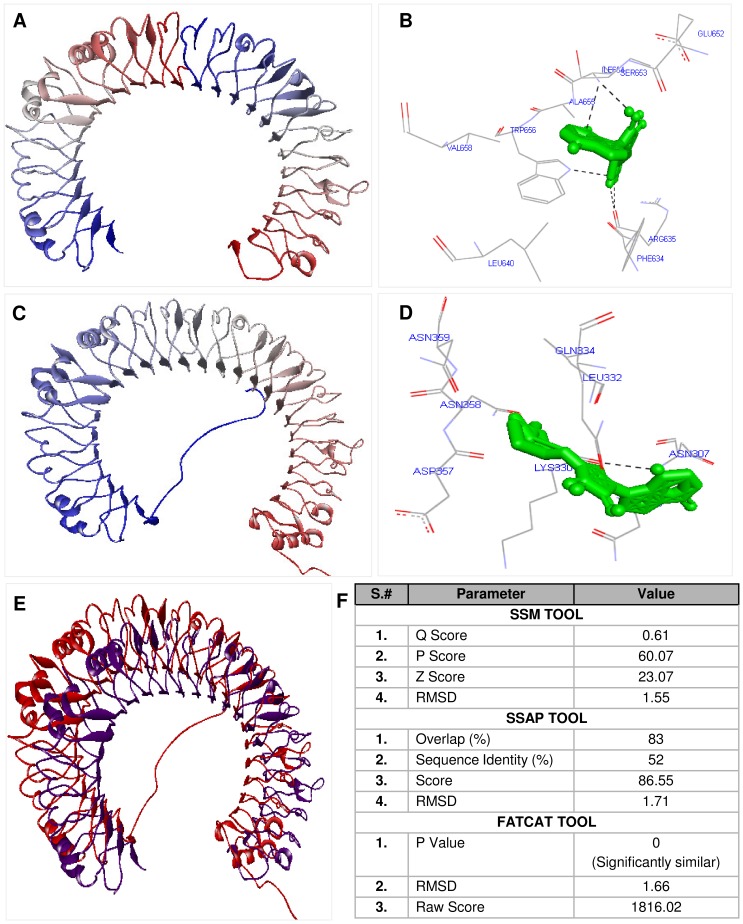
Homology modeling of shTLR3 ectodomain and docking with the synthetic dsRNA ligand poly I:C. The good model of human TLR3 (**A**) and shTLR3 (**C**) ectodomain that satisfies the various stereo-chemical constraints (predicted by atleast 4 models); **E** - The overlapped structure of both human and shTLR3; **B & D** – reveals the binding of the human and shTLR3 respectively with poly I:C reveals the difference in the number of hydrogen bonds and interacting amino acid residues.

The modelled shTLR3 protein was validated using Procheck Ramachandran plot (96.5%), ERRAT (58.509), Verify 3D (78.95%), What if server (−0.560) and ProQ (4.886). Among these five tools, 4 tools predicted the modelled protein to be a good model by satisfying the various stereo-chemical constraints (Figure 9A, 9C & 9E). Comparison of shTLR3 modelled protein with the crystallized structure of human TLR3 ECD was done with different tools like FATCAT, SSM, SSAP (Figure 9F) and it was found that the RMSD value was 1.71 (Figure 9F). Crystallized human TLR 3 ECD structure was docked with Poly I:C (synthetic analog of dsRNA) using Autodock Vina 1.1.5. revealed Poly I:C to bind by 5 hydrogen bond and interacted with 9 amino acid residues (Leu 640, Val 658, Trp 656, Phe 634, Arg 635, Ile 654, Ala 655, Glu 652, Ser 653) of human TLR 3 while shTLR 3 modelled protein was also found to bind with Poly I:C with a single hydrogen bond and interacted with 7 amino acid residues (Asn359, Asn 358, Asp 357, Lys 330, Asn 307, Leu 332, Gln 334). The binding energy for both the docked complex was found to be −7.2 kcal/mol which indicate sufficiently stronger binding.

**Figure 10:**
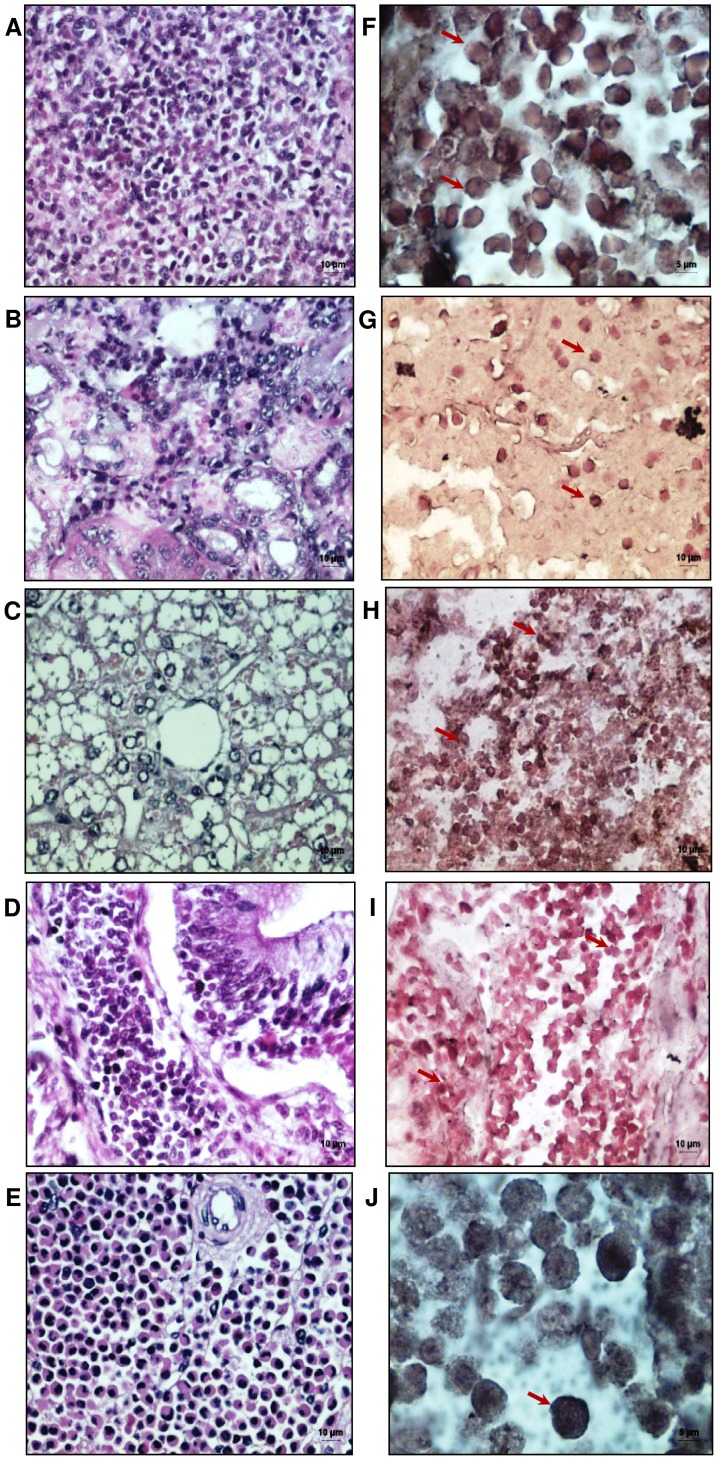
Localization of the shTLR3 by in-situ PCR in tissues sections of *Chiloscyllium griseum* spleen, kidney, liver, intestinal spiral valve and epigonal tissues. **A to E**: H & E staining of the above tissue sections; **F to J**: *In-situ* PCR revealed positive signals (brown areas due to the DAB substrate) in all the tissue types examined for TLR3 mRNA that can be localized to lymphoid cell types with thin rim of cytoplasm G: the tubular epithelial cells in the kidney revealed the presence of shTLR3 mRNA.

Expression profiling of the basal TLR3 mRNA levels by qRT-PCR in various tissues revealed lower levels in spleen and higher levels in kidney, ISV and epigonal (known to have immunological role). Microscopic examination of the H & E stained sections of the *C. griseum* spleen, kidney, liver, intestinal spiral valve and epigonal organ (Figure 10A to 10E) helped to establish the distribution of the different cell types prior to *in-situ* PCR. Localisation of the TLR3 by *in situ* RT-PCR revealed positive signals (purple colouration) in all the tissue types examined (Figure 10F to 10J). The TLR3 mRNA could be localized to lymphoid cell types with thin rim of cytoplasm except in the in kidney where the tubular epithelial cells showed considerable expression of TLR3 mRNA (Figure 10G).

**Figure 11:**
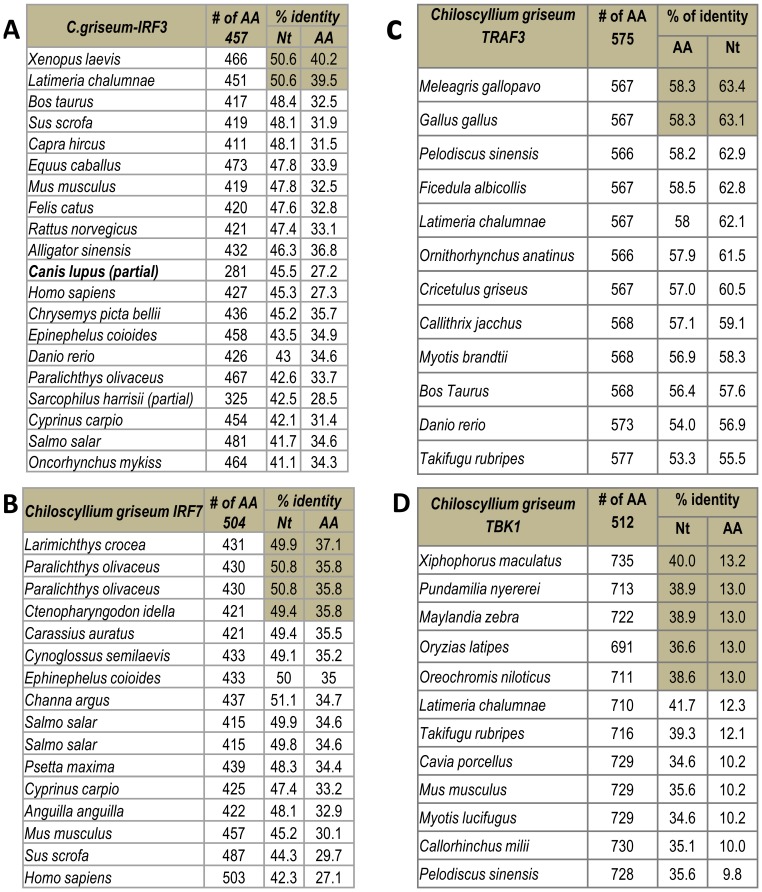
Percentage of nucleotide and amino acid identity of the *Chiloscyllium griseum* Toll-like receptor 3 (shTLR3) signaling pathway mediators with sequences of other species. The full-length coding sequence of the *C. griseum* TRAF3 (1728 bp), TBK1 (1529 bp), IRF3 (1374 bp), IRF7 (1515 bp) and Mx (partial 353 bp) sequences was aligned with the other sequences representing these signaling molecules types from different lower and higher order species using Clustal W in the Megalign Module of Lasergene (V 7.1.0). ***Note*** *: These signaling molecules revealed extreme diversity and the identity revealed close relationship with the higher vertebrate TLRs rather than the teleost. The TBK1 molecule was divergent among the signaling mediators*.

With respect to the downstream mediators of the TLR3 signaling pathway we could identify the full-length TRAF-3 (4672 bp with a predicted ORF of 1728 bp coding for 575 amino acids) from transcriptome and BLAST analysis revealed 53 to 58.5% amino acid (aa) identity to various vertebrates (avian species). The downstream signalling mediator of TRAF3 is TBK1 and the annotated shTBK1 was 1529 nt long coding for 512 aa which BLAST matched to *Ceratotherium simum simum* (44.3% aa identity). TBK1 mediates the signals either through IRF3 or IRF7. The IRF3 coding sequence was 1374 nt with a predicted protein of 457 aa long and more related *Xenopus laevis* with an identity of 28.5 to 40.2% at aa level with different species. The annotated IRF7 sequence was 1515 nt long that could be translated to a 504 aa protein which was more related to *Channa argus* and revealed 27.1 to 34.7% aa identity with different species compared. We could also show the presence of a partial transcript in *C. griseum* transcriptome that was 353 nt long which could be translated to a 117 aa peptide that BLAST matched with Mx protein (Figure 11) (Data S1).

### 7. Transcript validation

**Figure 12:**
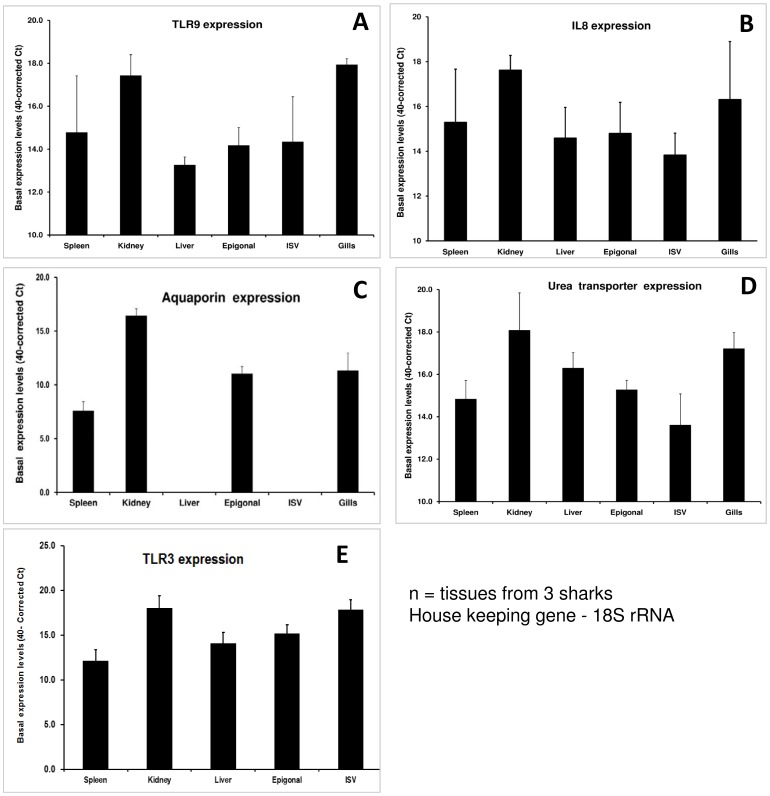
Transcript validation by real-time quantitative PCR. The basal mRNA expression levels of the identified transcripts were determined by designing the primers based on the sequence data generated. The expression levels of the different mRNA in the tissues are indicated 40-corrected Ct values. **A.** TLR9 mRNA; **B.** IL8 mRNA; **C.** Urea transporter mRNA; **D.** Aquaporin mRNA and **E**- TLR3 mRNA. *Note: The increased levels of TLR9, TLR3 and IL8 in kidney, gills & epigonal confirming their immunological role, while kidney and epigonal expressed higher levels of aquaporin confirming their role in solute balance*.

Evidence point to the evolution of specific immune response from sharks and they have been a species of interest from immunologist point of view [6], [7], [8]. In addition, functional annotation of the transcripts to different KEGG pathways revealed immune system genes to be more common or highly represented. In this context, the transcripts (transcript ID's TLR9 - 3176873; IL8-3160377; TLR3 – 3207159; urea transporter - 3154975; aquaporin - 3182301) were putatively identified for experimental validation by qRT-PCR. The levels of TLR9 were higher in the gills followed by kidney, ISV and epigonal (40-corrected Ct values of 17.9, 17.4, 14.3 & 14.2 respectively). The cytokine IL8 expression was found to be higher in kidney followed gills, epigonal and ISV (17.6, 16.3, 14.8 & 13.8 respectively). The detected expression levels of TLR9 and IL8 were lower in spleen when compared to kidney which did not correlate with the FPKM values. With respect to TLR3 the observed FPKM values were 1.11 for Spleen and 2.55 in the kidney. Similarly higher levels of transcripts could be detected in kidney followed by ISV and epigonal (40-corrected Ct values of 18.05, 17.85 & 15.16 respectively) in our qRT-PCR than spleen. The levels of aquaporin and urea transporter were higher in the kidney (16.4 & 18.1 respectively) and gills (11.8 & 17.2) that do play a role in solute balance and correlated with the observed FPKM values that were higher in the kidney (12.7 and 19.09 respectively) (Figure 12).

## Discussion

Sharks are an interesting species from the immunologists' point of view and to draw parallels across mammalian evolution of genes. Here we have used NGS to generate the ‘whole trancriptome’ from spleen and kidney of *C. griseum* and functionally annotated many interesting pathways that have not been reported earlier in sharks. Out of the 43,385 transcripts analyzed, we could identify 915 transcripts that matched to *Callorhinchus milli* and also 11 sequences that matched to *C. griseum* (out of the 13 present in the database) (as of September 2013). Two of the sequences reported in the database from *C. griseum* could not be detected in our transcriptome probably because of the smaller size of the reported protein sequence. This served as an ‘internal validation’ for the generated sequence data. Further, we also hypothesized that most of the assembled unigenes in our study have not been sequenced or reported earlier.

Functional annotation and GO classification helps us to obtain functional classification, distribution and also understand the physiological role. Furthermore, KEGG classification provides us with valuable information to generate interesting data that can further research on genetically and biologically complex pathways in the species of interest [27]. Among the main KEGG categories to which the transcripts mapped, the signal transduction and immune system related genes were highly represented in the transcriptome data from spleen (Figure 4B & 4C and figure S3A, S3B & S3C) while the kidney showed higher levels of transcripts relating to amino acid, carbohydrate and lipid metabolism and xenobiotic biodegradation.

Osmoregulation is one of the major adaptations in marine animals. We could identify the already reported UT gene and phylogenetic analysis revealed maximum identity (87 to 92%) with the shark UT gene (*Scyliorhinus canicula* &*Triakis scyllium* respectively) and its expression levels being higher in kidney and gills along with aquaporin suggesting their role in osmoregulation. We could also identify higher levels of the Megalin in kidney that has been shown to play a role in lipoprotein metabolism. In addition these receptors in kidney help in the uptake of the complex between vitamin D-binding protein and steroid 25(OH) vitamin D3 and hence a potent regulator of calcium homeostasis and bone turnover [32].

Sharks even-though being primitive in their evolutionary tree is known for their innate as well as a developed adaptive immune system similar to that of the higher vertebrates that are well known for its specific response, memory and versatility [33]. In this context, it is interesting to note that we could identify transcript matches to interesting innate immune receptor pathways and their downstream molecules in the transcriptome generated from *C. griseum*. The pattern recognition receptors (PRR) are an important range of innate receptors which recognize group specific pathogen-associated molecular patterns (PAMPs) that are exclusively present on microbes such as viruses, bacteria, parasites and fungi. The TLRs, NLRs and the RLR family play central roles in inflammasomes and autophagy through the induction a wide variety of cytokines/chemokines. Interestingly, some of the innate immune receptors like the toll-like receptor (TLR), nucleotide-binding oligomerization domain receptors (NLRs) and RIG-I like receptors that form a part of the pattern recognition receptors (PRRs) which have not been shown earlier in elasmobranches could be identified. Although NLRs and RLRs are recent additions to our knowledge on innate immune system, it has been shown that they are present in the primitive cartilaginous fishes, the sharks adding credence to the theory that these receptors are highly conserved across species. It may also be interesting to speculate that their biological functions could also be similar, so also their ligands. Although conserved, sufficient variability in the expression levels [34] and ligand binding [35] and downstream cytokine expression levels have been shown in farm animals.

The TLR types identified through the transcriptome include the TLRs – TLR2, TLR3, TLR6 and TLR9. Strikingly except TLR2, the other TLR types are intra-cytoplasmic in location and recognize endogenous ligands. To fortify the innate immune recognition pathway mediated by TLRs, we could also show the presence of the other known intra-cytoplasmic PRRs (such as NOD-1, NOD-2, NALP3, NALP-5, RIG-I and MDA5). The presence of the higher levels and the range of intra-cytoplasmic PRRs may aid to hypothesize that the shark cells are very difficult to parasitize by intra-cellular pathogen unless it has the arsenal to overcome the innate immune responses mediated by the above PRRs. At this stage it is unclear that whether the other TLR types homologous to higher vertebrate TLRs such as TLR4, TLR5, TLR7, TLR8 and TLR10 are present in sharks. Going by the depth of the sequencing and the extent of the reads generated, it seems that the TLR types even if present are expressed at very low basal levels (less than <1RPKM). In this connection, our earlier work on identifying TLRs based on PCR approach could also recognize only TLR2 and the reason could be due to higher nucleotide identity (55 to 72%) of TLR2 with other reported species. However, attempts to amplify the other TLR types using a degenerate primers or primers designed based on the conserved regions were not successful since the higher divergence of sharks TLR types identified in our study (TLR3, TLR6 and TLR9) with other compared species.

In addition to the toll-like receptors we could also identity the components of the classical TLR3 signaling pathway namely TRAF3, TBK1, IRF3, IRF7 & Mx as reported in higher vertebrates. Sequencing and analysis of the nucleotide sequence of the TLR3 revealed ∼70% nucleotide identity and only 66.6 to 67.9% amino acid identity with that of higher vertebrate TLR3 respectively. The top hit species sequences matches were to *Gallus gallus* and not with the teleost fishes and hence could could be one of the reasons for our failure to amplify the TLR3 of shark using the PCR approach in our earlier studies (data not shown). Hence, it appears that the TLR3 of bony and cartilaginous fishes may have evolved independently.

The presence of the TLR3 in the different organs of *C. griseum* could be shown in both immune and non-immune cells through *in-situ* RT-PCR approach. Although it would have been ideal to confirm the presence of this receptor at the protein level, the same could not be accomplished due to lack of specific antibodies and it is also very unlikely that antibodies to TLR3 from other species could have cross reacted due to the high divergence of the TLR3 protein. So far TLR3 basal mRNA expression levels has been reported in various tissues like kidney, liver, spleen, etc. in number of fish species [36]–[38]. In the kidney we could localize the TLR3 mRNA to the epithelial cells in the kidney as shown in earlier reports [39]. The localization of the TLR3 mRNA by in-situ PCR and the basal levels of this receptor validate both the annotation and sequencing. The qRT-PCR results also confirmed the higher expression levels of the TLR3 mRNA in kidney, ISV and epigonal as compared to the levels in spleen. ISV, epigonal and kidney have been shown to be important immune organs in sharks in contrast to higher vertebrates. The expression levels TLR across different organs has been shown to be an important determinant of innate resistance that could have accounted for these differential levels in the various tissues [40].

Although our evidence on the presence of TLR3 signaling mediators have been confined only to the genomic presence the fact that 5 major downstream mediators among the 6 involved in classical TLR3 signaling pathway in higher vertebrates makes us hypothesize that the same pathway could be functional in sharks. To add credence to this hypothesis the shark TLR3 modelled using human TLR3 crystal structure as a template could also be docked to Poly I:C with similar affinities as that of human TLRs (−7.2 kcal/mol) albeit with different number of hydrogen bonds [41].

In addition, some of the intra-cytoplasmic receptors identified in our transcriptome work showed a high homology with the higher vertebrate species such as MDA5 and NOD-1 (58 to 65% nucleotide identity with *Homo sapiens*). The lower similarity of NOD-2 and RIG-I (39 to 38% respectively with *Homo sapiens*) and some of the TLR types indicate that these receptors have evolved much from the primitive shark species. This is the first report for the genomic presence of these intra-cytoplasmic receptors in a primitive species. The present knowledge available indicating the presence of these intra-cytoplasmic receptors in higher order vertebrates and few fish species might prompt us to think that these receptors would have evolved later to fortify the innate immune defence mechanisms in these species only. However our finding confirms that both TLR and NLR have been present even in lower order species contributing to their holistic innate defence arsenal.

Although it would be interesting to confirm the biological activity of these receptors this can be taken up as a part of future studies. However, the genomic presence of mammalian orthologous of TLRs (TLR2, TLR3, TLR6, TLR9), NLRs (NOD-1, NOD2, RIG-I and MDA5) and the downstream signaling mediators (TRAF3, TBK1, IRF3, IRF7 and Mx) presents compelling evidence on the existence of the pattern recognition receptor mediate immune mechanism in a lower order vertebrate such as sharks. It might be hypothesized that although evolution of PRRs has occurred through gene deletion or duplication events, differential affinities and expression levels, our study proves that these receptors have been existing even from lower order invertebrates.

## Supporting Information

Figure S1 **Bioinformatics workflow for bioinformatics analysis of transcriptome data of** ***Chiloscyllium griseum*** **.**
(TIFF)

Figure S2 **The length and GC distribution of all assembled transcripts from spleen and kidney of** ***Chiloscyllium griseum*** **.**
(TIF)

Figure S3 **The differentially and highly expressed transcripts across the two organs (spleen and kidney) as assessed by Tag cloud plot.**
(TIF)

Figure S4 **KEGG annotation of the different pathways involving innate immune receptors and their downstream signaling molecules from the** ***C. griseum*** **transcriptome.** The *C. griseum* transcripts matching to the different components in the different pathways are ***highlighted in green*** **. S4A-** Transcripts matching to the different toll-like receptor (TLR) types and their downstream mediators and also the effector cytokines; **S4B** - Transcripts matching to the different NOD-Like receptors (NLRs) and their downstream mediators and effector cytokines; **S4C** - Transcripts matching to the different RIG-I-like receptors (RLRs) and their downstream mediators and effector cytokines and **S4D –** mediators of the chemokine signaling pathway.
(TIF)

Figure S5 **Phylogenetic relationship of the Urea Transporter transcript of** ***Chiloscyllium griseum*** **identified from the transcriptome data.** The sequence of the receptors urea transporter (276 nt) of *C. griseum* was aligned with the other sequences as listed in the supplementary ST1 from GenBank representing the UT receptor types from different lower and higher order species using Clustal W (codons) algorithm in MEGA 5.0. The identity of *C. griseum* UT ranged from 66.3 to 90.2% with that of other elasmobranch UT types reported with the maximum identity to 90.2% (with *Triakis scyllium*).
(TIF)

Table S1 **List of the GenBank accession numbers of the Toll-like receptor types, NOD1, NOD2, RIG-I, MDA5 and Urea transporter sequences used to determine the phylogenetic relationship of the sequence of** ***Chiloscyllium griseum*** **.**
(XLSX)

Table S2 **Sequence information on the primers used for real-time expression analysis.**
(XLSX)

Table S3 **Transcripts IDs of** ***Chiloscyllium griseum*** **with >300 bp long selected and used for the analysis in this study.**
(XLSX)

Table S4 **List of the assembled transcripts of** ***C. griseum*** **that were BLASTX annotated to NCBI.**
(XLSX)

Table S5 **Organism frequency table (species matches) based on BLASTX hit to NCBI database.**
(XLSX)

Table S6 **List of** ***C. griseum*** **transcripts with BLASTX top hit matching to ghost shark gene (** ***Callorhinchus milli*** **).**
(XLSX)

Table S7 **List of the assembled transcripts of** ***C. griseum*** **that were annotated to UniProt database.**
(XLSX)

Table S8 **List of** ***C. griseum*** **transcripts with GO annotation (Gene ontology summary).**
(XLSX)

Table S9 **List of** ***C. griseum*** **transcripts with eggNOG annotation.**
(XLSX)

Table S10 **Details of some immune transcripts identified in Spleen and kidney.**
(XLSX)

Table S11 **Details of some transcripts identified from kidney contributing to salt balance and excretion.**
(XLSX)

Table S12 **List of** ***C. griseum*** **transcripts that are differentially expressed at higher levels in spleen.**
(XLSX)

Table S13 **List of** ***C. griseum*** **transcripts that are differentially expressed at higher levels in Kidney.**
(XLSX)

Table S14 **Accession number of the sequences submitted to GenBank.**
(XLSX)

Data S1 **Sequence information, predicted open reading frame and the protein sequence of the dsRNA sensing C. griseum TLR3 and some important downstream signaling mediators (TRAF3, IRF-3, IRF-7, TBK-1 & MX).**
(DOCX)
