# Supplementary figures and images for: Transcriptome Profiling Reveals Higher Vertebrate Orthologous of Intra-Cytoplasmic Pattern Recognition Receptors in Grey Bamboo Shark
Source: PLoS One. 2014 Jun 23;9(6):e100018. doi: 10.1371/journal.pone.0100018 (PMC4067322; doi:10.1371/journal.pone.0100018)

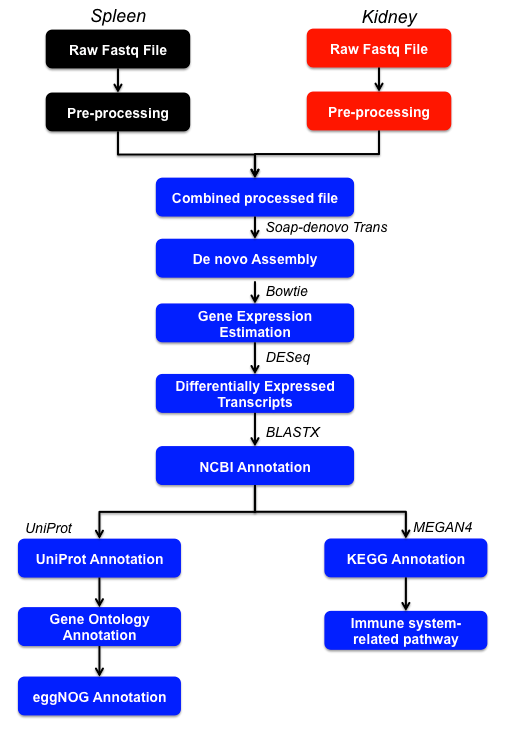

Supplement: Figure S1 — Bioinformatics workflow for bioinformatics analysis of transcriptome data of Chiloscyllium griseum . (TIFF) [file pone.0100018.s001.tiff]

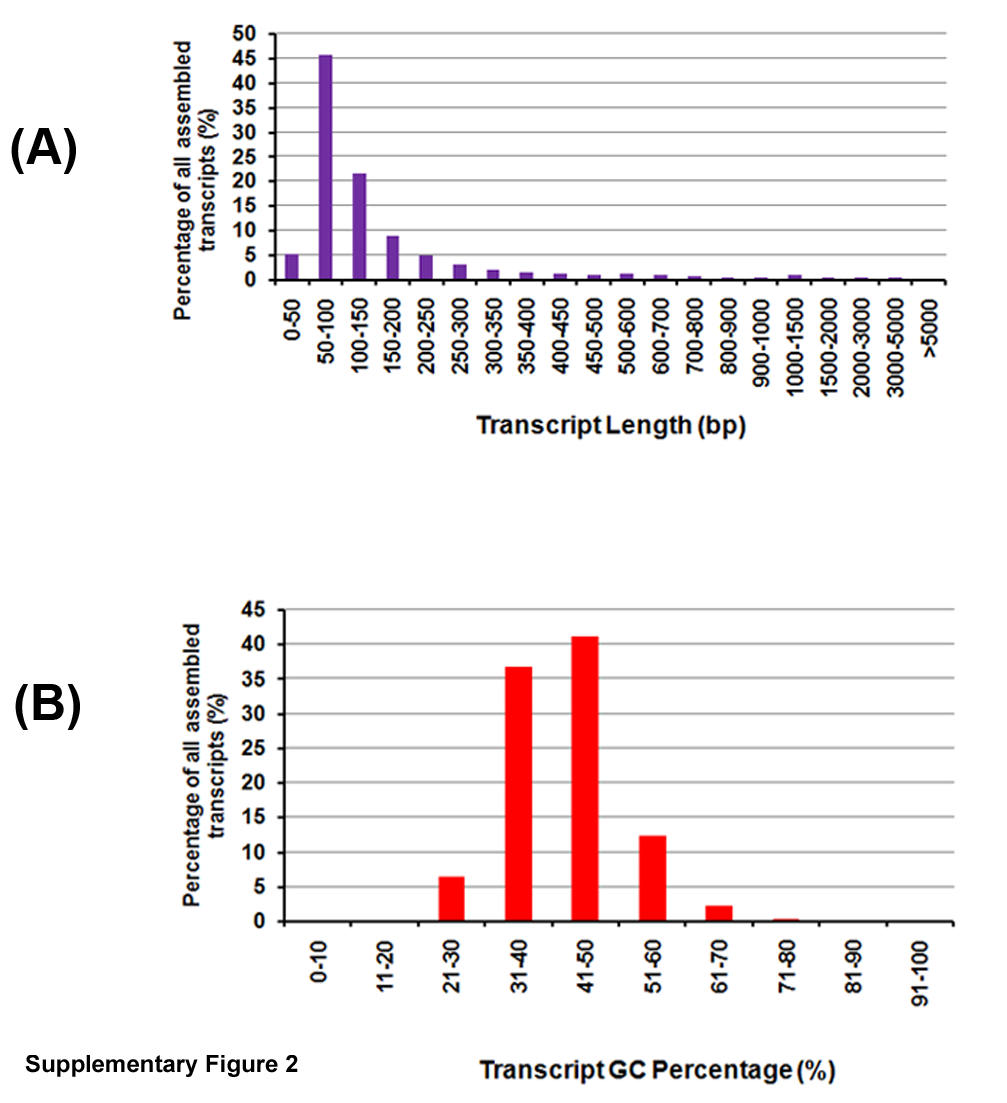

Supplement: Figure S2 — The length and GC distribution of all assembled transcripts from spleen and kidney of Chiloscyllium griseum . (TIF) [file pone.0100018.s002.tif]

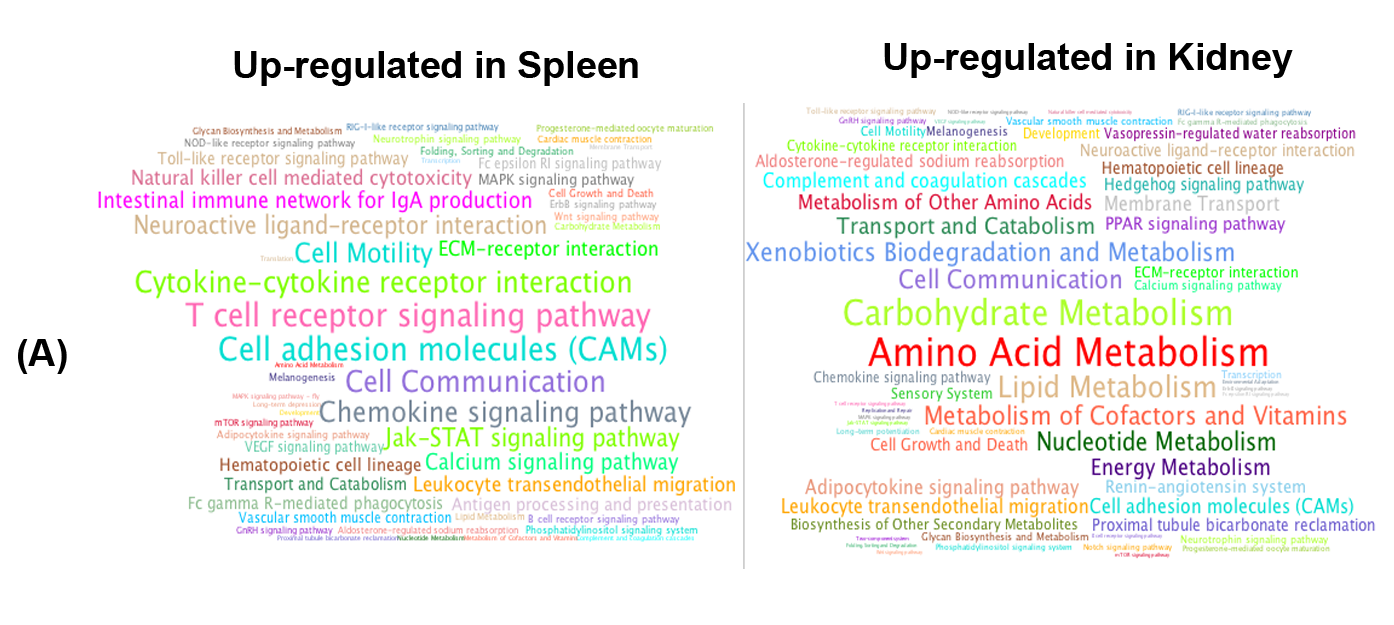

Supplement: Figure S3 — The differentially and highly expressed transcripts across the two organs (spleen and kidney) as assessed by Tag cloud plot. (TIF) [file pone.0100018.s003.tif]

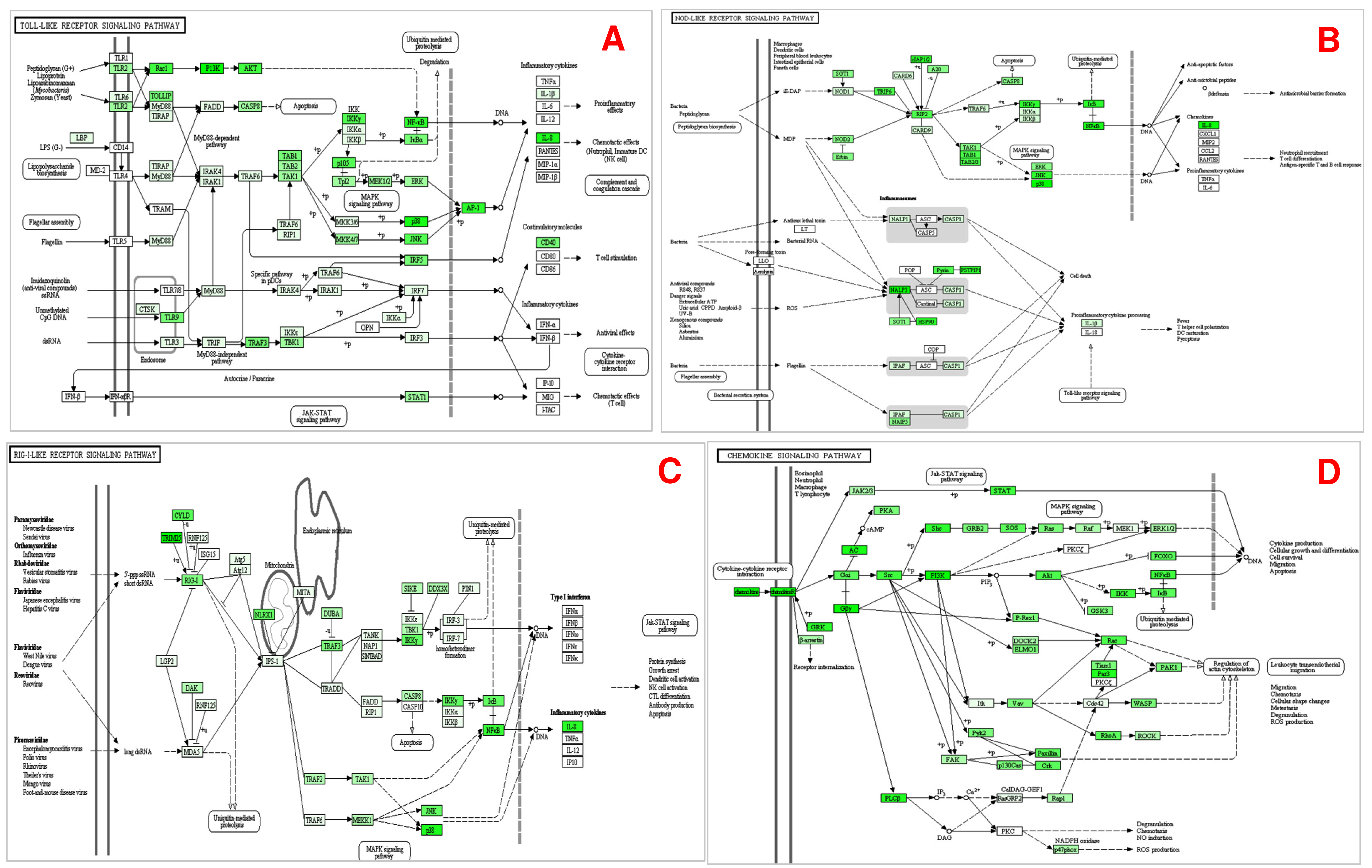

Supplement: Figure S4 — KEGG annotation of the different pathways involving innate immune receptors and their downstream signaling molecules from the C. griseum transcriptome. The C. griseum transcripts matching to the different components in the different pathways are highlighted in green . S4A- Transcripts matching to the different toll-like receptor (TLR) types and their downstream mediators and also the effector cytokines; S4B - Transcripts matching to the different NOD-Like receptors (NLRs) and their downstream mediators and effector cytokines; S4C - Transcripts matching to the different RIG-I-like receptors (RLRs) and their downstream mediators and effector cytokines and S4D – mediators of the chemokine signaling pathway. (TIF) [file pone.0100018.s004.tif]

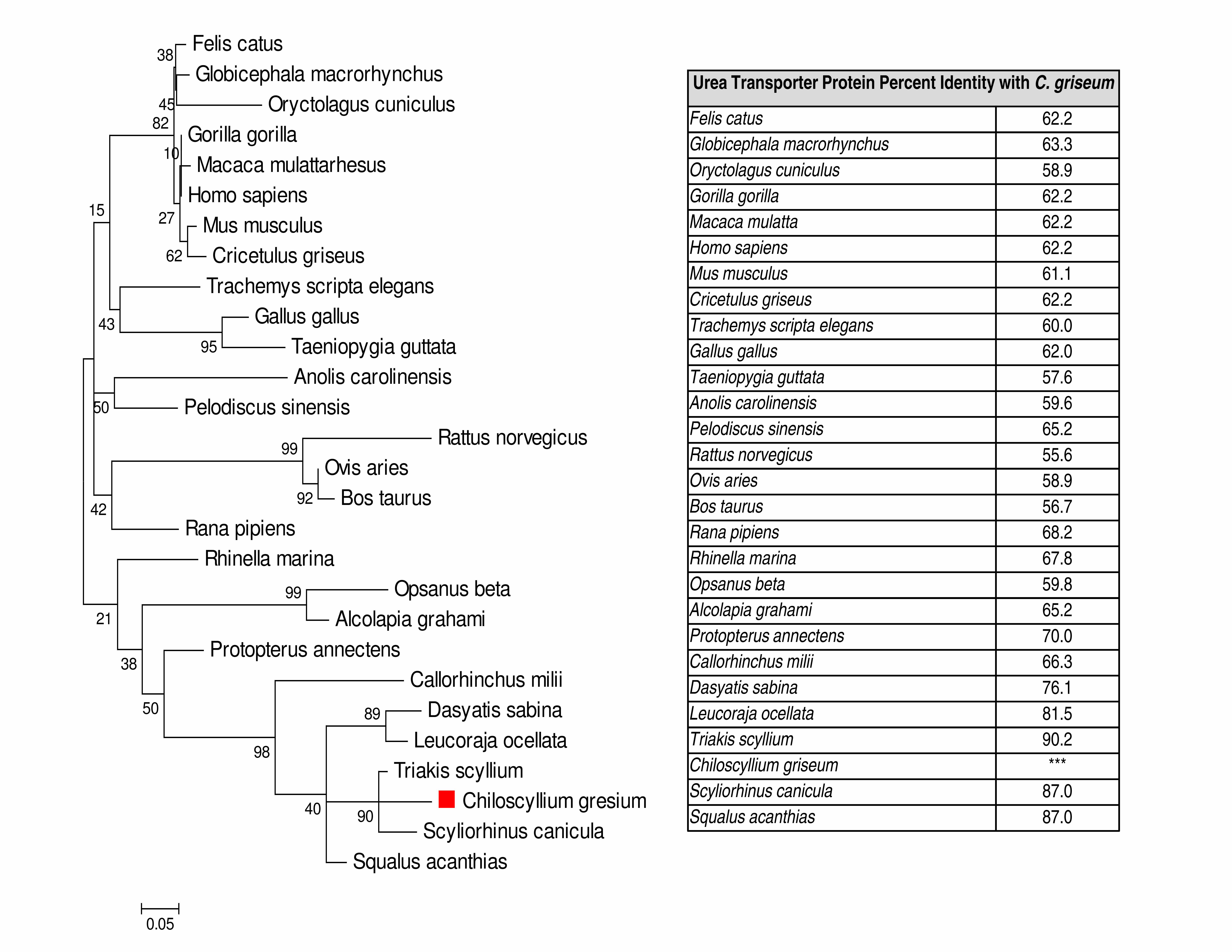

Supplement: Figure S5 — Phylogenetic relationship of the Urea Transporter transcript of Chiloscyllium griseum identified from the transcriptome data. The sequence of the receptors urea transporter (276 nt) of C. griseum was aligned with the other sequences as listed in the supplementary ST1 from GenBank representing the UT receptor types from different lower and higher order species using Clustal W (codons) algorithm in MEGA 5.0. The identity of C. griseum UT ranged from 66.3 to 90.2% with that of other elasmobranch UT types reported with the maximum identity to 90.2% (with Triakis scyllium). (TIF) [file pone.0100018.s005.tif]
