# Supplementary material for: Transcriptome Profiling Reveals Higher Vertebrate Orthologous of Intra-Cytoplasmic Pattern Recognition Receptors in Grey Bamboo Shark
Source: PLoS One. 2014 Jun 23;9(6):e100018. doi: 10.1371/journal.pone.0100018 (PMC4067322; doi:10.1371/journal.pone.0100018)
Supplement: Data S1 — Sequence information, predicted open reading frame and the protein sequence of the dsRNA sensing C. griseum TLR3 and some important downstream signaling mediators (TRAF3, IRF-3, IRF-7, TBK-1 & MX). (DOCX) [file pone.0100018.s020.docx]

**Data S1: Sequence information, predicted open reading frame and the protein sequence of the dsRNA sensing *C. griseum* TLR3 and some important downstream signaling mediators (TRAF3, IRF-3, IRF-7, TBK-1 & MX)**

**Chiloscyllium griseum TLR3 (2703 nt/ 900 aa)**

CTCTTCGAAAGTGAAAGTGAACAGAAGGGAAGCTGCCCAGGGTTTAGGTGCCTAAAATGAGCTGCGACCACAAACTGCCATCGCGCAGTGAACGAGACTCCGAATGGAGAACCGCGATAAACCAACTTCCCCTCAACGCCTGCACCAAATATTCACCTTGACTTGTAGGCACTGACGCGGTTGCACAATGT**CTGGACACGGGGAAATGAGAGAT**CAGTAACCTAAAGTTTGATGATATGAAACTGCAGCCAGTTTCGGTGATCCTTTGACCTTTTATTCAGAAGATTTCGGAAGCAGAAGAGGGACAGCTAATAAAAGACTGAAGGTGTTATATCTGAATGCTTGTGACGCAGATGTGAATAAAAGGTTGATTCCACATTTGAAGA

**5’ UTR**

**ATGAAAATGTATTTCCAGACTATCATCTTGTACTTTAAATTTGTGATATTATATCACGGTGCTACAGCCTCTGAAACTCAATGTAAAATACAGGGTCTGGTTGCGGATTGTAGACACTTAAAGCTCTCAAGCATCCCTTCAGACCTTCCAGTCAACATCACAGTGCTAAATCTCTCCCATAATCAGCTGAAGCACTTGTCAGCTTCTACACTGTCTCAGTACAGTCAGCTTCAAGTTTTGGATGCTAGCTATAATGTCATTACTAAAATCGAAGAAAACCTATGCAAAGTTCTTCCGTTCCTGCAAATGCTGGGGATTGAACACAATCAGTTCTCTCATTTGTCCGTACACTACTTCTCGTACTGCAGCAACCTGACTGAGCTGTACATACAGTTCAATCGAATCAAGGAGATCACAGGCAATCCTTTCACAAACCTTCAGGAATTGACGGTCCTGGATGTGTCTCACAATAAATTGGTTTCCGCCAAACTGGGAACTGAGCTGCAGTTGCAAAAGCTGCAAAGATTGGCGCTGTCTGCAAACAAAATCACACAGTTAAAGAATGACGATTTTGATTTCCTCAACAGCAGCACGCTGTACCAGCTGGATTTGTCGTCCAACAGTATCACTGAGTTTGAACTTGGTTGTTTCCACAAAATTGAAAGTTTACACAGGCTACTTTTGGACAATGTTGTTCTCCATTCCACTCTTGCTAAGCAACTCTGTGCAGCATTGTCACAGACTGACATTGCTGAGCTTTTTCTGAGGAACACCCACCTGAAAATACAAAACTCTACTTTTGCTGAATTAAGCAAAACAAACTTGACAACTCTTGATCTGTCAAACAATAAGTTGACTACAGTGCAAACTAATTCATTTCAGTGGTTGCACAGATTGGAATATCTTCAACTTGAAAACAACAGCATTCAACATGTCACCCCAAAAACATTTGCTGGACTGGAAAATTTGAGGTATTTGAACTTGAAGAAGGGCCTCAGCCAAGTAGAATCTCTCATTGATGATTACTCATTTCAGGAGTTGGGAAATCTAGTGGTACTCAATCTAGAAGACAATAATATTGCAAGTATAAGGGCACATACTTTCTCAGGCTTGAGAAATCTGAAATATCTTAGCCTGTATAAGTCATCAATTGTTGATCTAAAAACTATCAGTAACAAAACTTTCATGTCACTTGCAGAGTCTCCCCTTGTCAATTTAAATCTTACAAAAACAAAAATTTCAAAACTGCAGCCTGGAGCTTTTTCCTGGTTCAGGAAGCTGAAAAAGCTGGATATAGGTCTCAATTCCATCTCTCAGGTGCTAACAGGAGAGGAGTTTCGAGGTCTAAGTGAGGTAGAGGTGATATACCTGTCATATAACATGAAGTTGATTTTGTCTTCATCTTCCTTTATGCATGTCCCTTCTCTGAAAATCTTGATGCTCAGCAAATCAGCAATTTCCTCGTTGAATATTCAACCTTCGCCATTTGATTGTATTAAGAATCTCACTGTCCTTGACCTCAGCAACAATAATTTAGCCAATTTGGATAAGGATGTTTTCCGTGAGCTCCGGCAGCTTCAAGTTTTAAAGTTACAACACAATAACTTGGCACGGCTCTGGAAGAGTGTCAATCCTGGAGGTCCAGTTCTTTACCTAAGTGGTTTGAAAGAGCTTCAGATTCTGGATTTGCAATCCAATGGGCTCGATGAGCTCCCAGAGAAAGCTTTTCAACGATTGTATAAACTAAGTAATTTGGATTTAAGCATGAACAATTTAAACTTTCTTCCAGATGTTGTATTTAATGACTTGAAATCTCTCAAATCGCTTCAAATACAGAAAAATCTGATTACATCAGTTGAAAAAGATGTTTTTAGCCAGTTATTTAGTAACCTAACAGTTCTCTATATGGGATTTAATCCTTTTGATTGTACTTGTGAAAGTATTTTCTGGTTTGTTACCTGGCTGAATAAGACCAATAGCAGCATCCCTGGCTTGAACACTCAATACATCTGCAACACTCCACCAAGTTACCATAACCATTCTGTTGTAGCTTTTGACATATCTCCCTGCAAAGATGTTGCTCCTTTTGAGGATGCATTT**

**ORF**

**GTCATTAGTAGCAGCATTATACTTCTTGTCATTTTTGCTGTGTTTCTGATTCACTTTCACGGATGGTGGCTAGAATTCTATTGGAACATTTCTGTCAACCGAATATTTGGGTTTAAGGAAATAGACCATCAGGAAGCTCAGTTTCAGTATGATGCTTATATCATTCATGCCAAAAATGACATTAACTGGGTCAACAAATATTTGTTGCCTTTGGAGCAACAGGATCAAAAGACTACTTTCCAGTTTTGTTTGGAAGAAAGGGATTCTGAAGTTGGAATTTCTGAGCTGGAATCCATTGTGAACAGTATAAGCCAGAGCAGAAAAATCATCTTTGTTATGACTCAAGAGCTTTTAAAGGATCCCTGGTGCAGAAGATTCAAGGTACATCAGGCAATGCAGCAAGTCATTCAGCAAAGCCGAGATTCCATCATTTTGATATTTTTACAGGATATCCCTGATTATAAATTACACCAAATGCTCTGCCTCCGAAGGGGAATGTTCAAATCATACTGTATTCTAACCTGGCCAGCTCAGAGTAAGCGAATTCCTGCCTTTCACCAGAAACTTAAGGTCGCACTTGGTTCAACTAATCGAGTCCAATAG**

GCTTACATCTACTCACTGTCTGCACTTAGTACTCGGTCTAATACAAGGTTAAAATATAAAAAGGGAAATTTTGAGGCAAAAATCAGCAACAAAT**TCTGAACAAAATTATGAAGTGCT**CTCCTTTTGTGCTCTACCCATACCTCGTGTCACTCCTTCACATTTTCCCTGAAACCCTGCAAATCTTTTCTCTTCAGATAATGACCTTGTTCTCCTTTGAAACTATGTCCACCAGACTTTCAGGCTTTCAACAGCCTAACTCTCCATAGCGTAGAAACATTTTCCCTCGTGTTACTGTTGTCTCTTTTGCCAGTCACCTTAAATCGGTGCCTTCTAATTCTTGGTCCTTTCACCAGTGGAAACAGTTTCTTCTGACCTGCGTTGTCTAAATCCCTCTCATGACCCTGAGCACGTTTGTGAAATCACCTGTTACCTTTCTTCAAGGAGAAAAGTCTCAGGTTCTCCAATCAATCCACATAATTGGACGGCATAGTGGCTCAGTGGT

**3’UTR**

**Chiloscyllium griseum TLR3 protein (900 aa)**

***Signal peptide***

MKMYFQTIILYFKFVILYHGATASETQCKIQGLVADCRHLKLSSIPSDLPVNITVLNLSHNQLKHLSASTLSQYSQLQVLDASYNVITKIEENLCKVLPFLQMLGIEHNQFSHLSVHYFSYCSNLTELYIQFNRIKEITGNPFTNLQELTVLDVSHNKLVSAKLGTELQLQKLQRLALSANKITQLKNDDFDFLNSSTLYQLDLSSNSITEFELGCFHKIESLHRLLLDNVVLHSTLAKQLCAALSQTDIAELFLRNTHLKIQNSTFAELSKTNLTTLDLSNNKLTTVQTNSFQWLHRLEYLQLENNSIQHVTPKTFAGLENLRYLNLKKGLSQVESLIDDYSFQELGNLVVLNLEDNNIASIRAHTFSGLRNLKYLSLYKSSIVDLKTISNKTFMSLAESPLVNLNLTKTKISKLQPGAFSWFRKLKKLDIGLNSISQVLTGEEFRGLSEVEVIYLSYNMKLILSSSSFMHVPSLKILMLSKSAISSLNIQPSPFDCIKNLTVLDLSNNNLANLDKDVFRELRQLQVLKLQHNNLARLWKSVNPGGPVLYLSGLKELQILDLQSNGLDELPEKAFQRLYKLSNLDLSMNNLNFLPDVVFNDLKSLKSLQIQKNLITSVEKDVFSQLFSNLTVLYMGFNPFDCTCESIFWFVTWLNKTNSSIPGLNTQYICNTPPSYHNHSVVAFDISPCKDVAPFEDAFVISSSIILLVIFAVFLIHFHGWWLEFYWNISVNRIFGFKEIDHQEAQFQYDAYIIHAKNDINWVNKYLLPLEQQDQKTTFQFCLEERDSEVGISELESIVNSISQSRKIIFVMTQELLKDPWCRRFKVHQAMQQVIQQSRDSIILIFLQDIPDYKLHQMLCLRRGMFKSYCILTWPAQSKRIPAFHQKLKVALGSTNRVQ.

**Chiloscyllium griseum TRAF3 (1728 nt/ 575 aa)**

**>C.griseum TRAF3**

ATGGCAACTGCAAGAAACATAGAGTCATCGGCCCCACAATTTTCCCTGATGCAGCAGACCAATGTGGCTAATGTTCCAAATAGCAGGCTGTCATCTCTAAGCATTGAACCAGACGATGGGGGCTTCAAGGAGAAGTTTGTGCTGAAGCTGGAAAACAAGTACAAATGTGAATATTGTCATCTTGCACTGTGCAATCCCAAACAGACTGAATGTGGCCACAGATTCTGTGAAAGTTGTGTAAGGAAAATCATTGGGAGTCCGAAACCAGTTTGTCCAGTTGATAATGTGCCTTTATTTGAGAATACGATTTTCAAGGACATCTGCTGTAAAAAAGAAGTTCTTGCACTTCACGTCTTCTGTAGGAATGAGATAAATGGCTGCAAGAAACAGCTACCTCTTGGAAATCTTGAGGGTCACTTACTTGAATGTCCTCACCAGGAAGTCCGCTGTGCACGCAGGGGATGCACAGAAATGGTGCAAAGGAAAGATCTAGCTGACCATTTAAATTCAAGCTGTAAATATCGGGAGCAAACCTGTAAATACTGCAATAATGAAGTTGCTATTGCTGAGTTAAATAAACATGAAGAATTTGACTGTCCGTTATTCCCTGTGCCATGTCCCAATAAGTGCAATGCTCGTATTCTGAGGGGAGAGTTAAATAGTCATCAGTTGCAATGTCTCAATGTGGTCATGACTTGCCCTTTCTCTAAGTATGGATGCAATTTCCAAGGAACCAACCAGCAACTTAAAATGCATGAAGCTGATAGTATGGCTCAACATTTGAACTCTGTGTTGATGAAGAATGGAGATCTCGAAAATACGATTTTTGAACTTCAGAACAAGTTGCAGGAAAAGTCTAAAATGATTGATATTATGTCAGCTCAGATAAGCCACTTGGAGAAGGAACAGAGTAAATTAGCACAGCTTGCAACCAAAAATGAAAATCGACTTGGATACATGCAGAAAATGTTAGCTAGTCAGACAGATAAGTTGATGAATATTGATCAAGCATCGCAACAGACTTATCAGAAGCAGGAGGAGTCTGTGAGAGAGGTGAAAACTTTGGAAGAATCTGTAGACAAGCTCCAAATCCAAATCCGACAGCTGGAACTAGCGGGCAGAGCTGGTGGGATGGCCACGGGAGCAGCAAACCTGAGTGCATTGGATAACCAAGTAAAAAGACATGGCAGCCTTCTCAGTGTACATGATGTGCGCTTAGCAGATATGGACCTACGCTTTCAAGTGCTCGAAACGGCAAGCTACAATGGAAAATTAATTTGGAAAATCCGAGATTATGCACGCCGAAAACAGGAAGCTGTTAGTGGAAAAACATTGTCCTTGTATAGCCAGCCATTTTACACTGGATATTTTGGCTATAAGATGTGTGCTCGGGTTTACCTCAATGGAGATGGCATGGGCAAAGGAACTCACTTGTCGCTGTTTTTTGTCGTTATGCGGGGAGAATATGATGCACTGTTACCGTGGCCCTTTAAACAGAAAGTAACTTTGATGTTATTAGATCAAGCACCGACAAAAAGCCATTTAGGTGATGCCTTTAAGCCAGATCCCAACAGTAGTAGTTTTAAGAAACCAGTGGGAGAGATGAATATTGCTTCAGGTTGCCCACTCTTTGTTGCACAGACTGTTTTGGAAAGTGGGACGTATATTAAAGATGACACTATCTTCATAAAAGTAGTTGTTGATACCTCAGATTTGCCAGATCCATGA

**>C.griseum TRAF3 protein (575aa)**

MATARNIESSAPQFSLMQQTNVANVPNSRLSSLSIEPDDGGFKEKFVLKLENKYKCEYCHLALCNPKQTECGHRFCESCVRKIIGSPKPVCPVDNVPLFENTIFKDICCKKEVLALHVFCRNEINGCKKQLPLGNLEGHLLECPHQEVRCARRGCTEMVQRKDLADHLNSSCKYREQTCKYCNNEVAIAELNKHEEFDCPLFPVPCPNKCNARILRGELNSHQLQCLNVVMTCPFSKYGCNFQGTNQQLKMHEADSMAQHLNSVLMKNGDLENTIFELQNKLQEKSKMIDIMSAQISHLEKEQSKLAQLATKNENRLGYMQKMLASQTDKLMNIDQASQQTYQKQEESVREVKTLEESVDKLQIQIRQLELAGRAGGMATGAANLSALDNQVKRHGSLLSVHDVRLADMDLRFQVLETASYNGKLIWKIRDYARRKQEAVSGKTLSLYSQPFYTGYFGYKMCARVYLNGDGMGKGTHLSLFFVVMRGEYDALLPWPFKQKVTLMLLDQAPTKSHLGDAFKPDPNSSSFKKPVGEMNIASGCPLFVAQTVLESGTYIKDDTIFIKVVVDTSDLPDP.

**Chiloscyllium griseum IRF-3 (1374 nt/ 457 aa)**

**>C.griseum IRF3 nt**

ATGGGTTCCCAGAAGCCGTTGTTGTGTGATTGGCTTATTGAGCAGATAAACAGTGGGCTGTACCCCGGCCTCTCTTGGCTGAATGCGGAGAGAACTCGATTCCAGATCCCCTGGAAACATCGCTCGAGGCACGACATTTGTGAAGATGACTTCAAAATATTTGAGGCTTGGGCGATAGCAAGTGGCCGATATAAGCCAGGGATTGATGTTCCTGACCCTATTGTATGGAAGAGAAACTTTCGCAGTGCTCTGAATCGGAAGAAGCATTTTCGCCGTGTGGTTGACAATAGGAATAATTCTGAGAGACCTCACCTGATTTTTGAGATCCAGAGCATTGGCCAGCAGAGTGGGGCCAGTGCGGAGGAGGAGAGTGAGGAAGTCAGTCCTGCCATCAACAGCGCCAGCCCTGGACTCTCCACGGGCTCATCCCCAAACGTGCAGGGGACCCTGGAGAGCAGTTTGCGGGACATGACCATATTCGACTGGGAGTCAGGGGGCGGTGGAGAAGGAACCATGATGGAAGTGGGAGCAGCCCTGTATCCTTCCGACTATGCTCCAGTCTATACTGTGGCTCCAAACAACCTAAGTCAAATAGACCCCAGTGGCCAGCCCACTGCCGGTGCAGATGCTGCCTGTGCGATGCCTCTTGCAGTCGAAGGTCCCAACATGGTCATCCCAAACGTTGGCGAGTTTCAAACACAGATGAGGGGCTATTTCCAGAATGGACACTTCGCAACCGAGTTTGAGATAACAATCTACTATCGTGGCAAGAGGGTGAAGGAGCAGACTTTGAAGAACACCAATGGATTCCGACTGTTTTACACATCGGAATCTAAATTCCCGTACCTAGAGGACCTCCAGTTCCCAGAAGTAGCCTCCTGTTTGACTGACCAGCAACAAATCATGTACACAAATGTATTGTTGGAGAGGATGGGCCAAGGCCTGACCGTGGAAGTGAACAATAATCAGATCTACGCACAGAGACACGGGAGCTGTAGGGCTTTCTGGTCAATGACGGAGAACCCCAGCAGTAAGGAACCTCGACAGATCTCCAGCAGGGAGCTCACTGTCCTGTATGACCTGCCCCAGTTTCATCAAGAACTGTGTGCCTTTCTGAACTCTGAGAGTGGCTCTCCGCAGTACTCGATTTGGCTATGTTTCGGGGAGCTCTGGCCAGACTGTGGTGATAAACCCTGGAATAAGAAGATGATCATGGTGCAGGTGACACCTATAACCTTCAAGCTTTTACATGAACTGGCACACGGAGTGGGAGCCTCCTCACTGAAGGATGATTCTATAAACCTCCAGCTCTCTGACCCCCTCTCCTCCTCCAGCCTCCTGTCCATACTGGAACAGTGCATGGATATCGAGTAA

**>C.griseum IRF3 protein**

MGSQKPLLCDWLIEQINSGLYPGLSWLNAERTRFQIPWKHRSRHDICEDDFKIFEAWAIASGRYKPGIDVPDPIVWKRNFRSALNRKKHFRRVVDNRNNSERPHLIFEIQSIGQQSGASAEEESEEVSPAINSASPGLSTGSSPNVQGTLESSLRDMTIFDWESGGGGEGTMMEVGAALYPSDYAPVYTVAPNNLSQIDPSGQPTAGADAACAMPLAVEGPNMVIPNVGEFQTQMRGYFQNGHFATEFEITIYYRGKRVKEQTLKNTNGFRLFYTSESKFPYLEDLQFPEVASCLTDQQQIMYTNVLLERMGQGLTVEVNNNQIYAQRHGSCRAFWSMTENPSSKEPRQISSRELTVLYDLPQFHQELCAFLNSESGSPQYSIWLCFGELWPDCGDKPWNKKMIMVQVTPITFKLLHELAHGVGASSLKDDSINLQLSDPLSSSSLLSILEQCMDIE.

**Chiloscyllium griseum IRF-7 (1515 nt/ 504 aa)**

**>C.griseum IRF7 nt**

ATGAACAACCATAAACCACAGTTCCGATCCTGGTTAATCGCTCAGGTTAACAGCGGTAGTTACCATGGGCTGCGCTGGCTGAACCACGAAAAAACCATGTTCCGAATTCCCTGGAAACATGCCGGCAGGCAGGACCTCTGTGAGAATGATTACAGCATCTTCAAGGGTTGGGCAACAGTGAGTGGGAAATGTACTGATGGTCCACCCAAATGGAAAACTAACTTCCGATGTGCCCTCAACAACATTGACTCATTCATTTTGCTGGAGGACAACTCGAAGGAATCAACGGATCCACATAAAATCTATGTCATTACTAATGACAAGGTCAATTCAGCGCTTGATCCAAACATGGATGGAACTCTGCAGGAAGATGATAACCTCGAATCACAACTGCACATCAGTCCTGAGACTAACCTGGAACAGCCTAACTTTGCACTCTATGATACCCAGCAGTCCTCTGCAATACCCAACACTGAGGGACAGTTAGAAGAATGGATTCCAGATTTTGATTTAATGAATCTGTCGGACAGTGCTGAAGACCAAATGGCTCCTGGCCTGTTCAATCCAGCAAACATCTATAACTCAGCCATGAACAGAATCTACAATTTCCAGGTGCCTCCTGGCACTCCTCCCCAGGAAGTTTTCAATGTGTCACCAGCCCAAGTCGTGCCCACTGATGAGTACAGAGAGCTGCCAAATCAAACATTCAACGCAGACCAAACTTGGGAGCCTCTGCTTGTCCCAACATTTCATGTAATGGAGAATGTGGCCCAGGAGATACACTATCCTGCTTCAGATGTCCCTCTGAATCCAGCTCCAGGAATGCAGGTTGAAATTCCAATTGTATCTCAACTCCAACAAGTTCTAAGAGATTTTGACATTACCATTTACTACCGTGGCAAAAAAGTCTTTCAGAGCACGGTTAGCAATGCTAATGGCTGCCGCCTGTATCATGATGAAGAGAATGAAAGATTCGCTCAGCTACAACACATCAGGTTCCCAAGTACAGAGGAGATAAAGGATCATCAACAGAAAAAGTTCACCAATTGTCTCTTGTCAAACATGGCAGGAGGACTTCTTCTGGAGAATAAAAATGGAGATCTTTTCGCCAAGAGACTGGGCAAGTGTCAAGTCTTTTGGACCCGTTCAGGGGCAACCATGAATGAGGAGTCACAAAAGCTGAACCGAAATGAGGAGATTAAATTATTCAGCCTGAAAGATTTTTACACGGAATGTATGGAGTTCATGGAGCAGCAACGTGGAATGCCACAAAGCTCCATCTTCCTATGCTTTGGACAACAATTCCGGATAGGAAATGAGAAGAAGAAACTCATCCTGGTCAAGATTGTTCCTAAAATCTGCATCTGTCTGATTGAATACACCCATCAAGAAGGAGCATCCTCACTGACTAGTGATAATGTCAGCCTGCAGATATCTAATAATTCCAGCACTTCATCCATGGAGAACTTGATGGCTTTAATCCAAGAACTGGAAAACCTGATGGAGCTAGAGTAG

**>C.griseum IRF7 protein**

MNNHKPQFRSWLIAQVNSGSYHGLRWLNHEKTMFRIPWKHAGRQDLCENDYSIFKGWATVSGKCTDGPPKWKTNFRCALNNIDSFILLEDNSKESTDPHKIYVITNDKVNSALDPNMDGTLQEDDNLESQLHISPETNLEQPNFALYDTQQSSAIPNTEGQLEEWIPDFDLMNLSDSAEDQMAPGLFNPANIYNSAMNRIYNFQVPPGTPPQEVFNVSPAQVVPTDEYRELPNQTFNADQTWEPLLVPTFHVMENVAQEIHYPASDVPLNPAPGMQVEIPIVSQLQQVLRDFDITIYYRGKKVFQSTVSNANGCRLYHDEENERFAQLQHIRFPSTEEIKDHQQKKFTNCLLSNMAGGLLLENKNGDLFAKRLGKCQVFWTRSGATMNEESQKLNRNEEIKLFSLKDFYTECMEFMEQQRGMPQSSIFLCFGQQFRIGNEKKKLILVKIVPKICICLIEYTHQEGASSLTSDNVSLQISN

NSSTSSMENLMALIQELENLMELE.

**Chiloscyllium griseum TBK1 sequence (1520 nt/ 512 aa)**

**>C.griseum TBK1 nt**

GTCCCTGTTGACCTACTGCT– 5’ UTR

**CDS:**

**ATGGATTCATTCAACTATGAAGACGACATCAGCATCTTGACACAAAACGGGCAGCAGGACGATACAGAGTGGGCTGATGCACCAAATACAGACCTTACGGGCGAGGAATACTCCGCATCCCACTATGCACTCATCACTGCCTACAATGATATTAAACAGAGATTGATCGGCATGGAGAGAGATAACTCCACCTTGAAGAGAAAGCTCAGACAATATGATCTCAAGTTTTCCTTGTCAAATGAGCTTGTGGATGATAAGAAGTTGCCGCAGTCCTTGCTTGACATTACCTTGCTGAGAAATGAGAACACTCGGCTGAGGGAGCAGCTGGAGCACTTTAGAAACCAGCTTCGTGACTGTAAAGAAAGGGAGGATCACCTGGATGAGATCATTAAAGCTTATGAGAAAATACGTTCTGAGAAAGATGACCTACATCAGCAATTAGAAGAAATGACGGTGCTGGCCGAGGATCACATCTCCACGATTCAGAGCTTGGAGCAAGCTCTGAGGCTGAGGGACAGTTCAGTTCAGATACTCAATGATCAGCTCCAGGCAAAAAATGAACAAATTATCCAGCTGAGCCCAACACGTAGAAGTCCTTACGGGCTGGAGAGTCCCAGACACCAGCAGAACTGCAGGGTGGTCGATCCTCAGCTCGATGAGCTGGAAGTTCAGAGGTTGCAGGAAAAGGTGGACGAACTCCAGAGGAAGCTGCACAGTTGCCAGTGGCGGGAAAGACAGTACAAAGAGGAGTGCGACCGCTTACAGTCTCAGCTGAGCCAGCAGAGTCTACAGGAGAGTTGTGCACAGGAGCCAAGCCACGACCCCCATGACATGGAGTGGATAAAGAACACAGAAGAAGAACAGGAAAATCTGGTTCTGGCATACACTGAGCTGGCCCAAGAACTCTGCCAGTTACGGAGTCTGACTGAGGCTCAGACTGAGATCCTGAGGAGGCTGTCTGAGGAACAACTGACTAATAACGCCCATCTCCAGCCCTCTGGCCACGTGAGGCAAGCCGCATACTCCAGCTACCCCAGGAGCACGAGCCACCGTCTCCAGAACAACTTTCAAGGGTGCCGCAGTTACTCTGAAGTCAGTGATGGGAAGGTGGAGAGCCACACAATGCCATCTCGACTGCCAGCAGATGACCAAACCTCACCAACGCACAGGCAGTACCTTGCTAGTGATTACCTGAAGGTGCCTGACAGCCCAGACATTGGCCCATTCGAAAGGCAGATCGAGTCTGAAGATGAGGATTGGATGAACCATAGTCCTCCTGGCACTTTGGACAGGGGAATAAGGAGCACTTCCTCCTGTACAACGCTCCCAATCCCAGACACTACTATGAATAGGAGTTCGACCGAGTATTCCAGATCAGAACATGCGCAGTCATGGCCTTCTATCAATCTTTGGATGGAAACGGGAGACTCGGACATCAGGAGCTGCCCACTGTGCCAATTAGCCTTTCCCCTCAACTACCCAGATGATGCCCTCATAAAACACATTGATACTCACTTGGAAAACAGCAAGATCTGA**

TAGCACTGCTCCTCACTGAGTCGTGGAAGGATTGAAGAAAATGAAGAAGCAAAAGATTGTCTTGAAAAACTGATACAAGAGAGCAAATATTGCAGAACCAAAAAAGGAAGCAAATGTTCTACTTGCTAATCTAATTTTACTTTCTGTTGGGGTTGGAGGGAGATGACTATGTGTCTTGAATGGACCTAAGAAATAGATAGAAATGCTACTTTGAAGGCTGCTCCACATTTGAAGAATAAAGATAAACATTTTTGATCAGTTGCCGTCAAACGTGACAAATGATATGTCTTCCAGTGAAATCAGTTTACTTCCAAAAGCAGAATACTTGAAGCTTGTTCAATGCGAATCAGGATATGCTGGAATCAGTAGCTGGAGAATGAAGTTGTCCCATTGCAAAATGAAAAGGGGCAACCGAAAGGTTAGGTCTCGATGTTAAGTTAGGAAGTCTATTGCTTAACGGCGAAGATTGAACTGGTGTAGTACATTATTGGGATAGGTTTCCTGTGATACTTATACAGTTGGCTTCTAGTTATTGAGGTTTAACAAAAATATAATTTATTGTTGAGGAATGGGGAGTTGCAGAACATCTCATAGATTGATAATGCAGTCCCTAACCTGAAGGACAAGTGCATCAATGTCACAAATTGTCCTTCTCTCTTGTCCTCCCCCATTGATTCCAGGGTTGGAAATTCTACATCTGATATGGTTTTGAACACCTTGGTGAACGACAGTAGGAAATAAAACAAATTGAACTTATTAATATTGACTGTACACATGGTTCAAATTTATGTAAGGTTGTCACATGCATGTCTCTTATTCACATTTCTTGACATCTGACTCCATACACAGAAGCTGTTACAGGCCAGCATAAATTCAATCTCTATCTGTAACAGAATAAAAGAAATACTTGCATTTATATAGCACCTATA – 3’ UTR

**>C.griseum TBK1 protein**

MDSFNYEDDISILTQNGQQDDTEWADAPNTDLTGEEYSASHYALITAYNDIKQRLIGMERDNSTLKRKLRQYDLKFSLSNELVDDKKLPQSLLDITLLRNENTRLREQLEHFRNQLRDCKEREDHLDEIIKAYEKIRSEKDDLHQQLEEMTVLAEDHISTIQSLEQALRLRDSSVQILNDQLQAKNEQIIQLSPTRRSPYGLESPRHQQNCRVVDPQLDELEVQRLQEKVDELQRKLHSCQWRERQYKEECDRLQSQLSQQSLQESCAQEPSHDPHDMEWIKNTEEEQENLVLAYTELAQELCQLRSLTEAQTEILRRLSEEQLTNNAHLQPSGHVRQAAYSSYPRSTSHRLQNNFQGCRSYSEVSDGKVESHTMPSRLPADDQTSPTHRQYLASDYLKVPDSPDIGPFERQIESEDEDWMNHSPPGTLDRGIRSTSSCTTLPIPDTTMNRSSTEYSRSEHAQSWPSINLWMETGDSDIRSCPLCQLAFPLNYPDDALIKHIDTHLENSKI.

**Chiloscyllium griseum Mx sequence partial (353 nt/ 117 aa)**

**>C.griseum Mx nt**

CAGGAACCAAACAGGCAAACACATTCAGGACAGACAAACAGCATTATGCAAGCACAACACATACTAGCTGAGGTACCCACAACCAATGGTGAGAGTGCTGTGCCGATCTGTAGCCAAGGCTTTGCTAACTCCAAGGGGTCTACCTGTCAGAAGGACATCTGGAAGAGTGCATTTGGTGTGTTGGAGACACAAGAACAGAAGGAAGAGAGGAGAAAAACATCAATGAAGGCAATGGACACTGTGTTTTTCAATGAGTATGAAAGTAAAGTACGACCTTGTATTGATCTCATTGACCAACTGAGAGCTTTTGGTGTGGACAAAGATCTGGGACTTCCAGCCATCGCTGTGATTGG

**>C.griseum Mx protein**

QEPNRQTHSGQTNSIMQAQHILAEVPTTNGESAVPICSQGFANSKGSTCQKDIWKSAFGVLETQEQKEERRKTSMKAMDTVFFNEYESKVRPCIDLIDQLRAFGVDKDLGLPAIAVI
